# Integrated memristor for mitigating reverse-bias in perovskite solar cells

**DOI:** 10.1038/s41586-026-10275-3

**Published:** 2026-03-18

**Authors:** Mahdi Mohammadi, Fuxiang Ji, Tristan Sachsenweger, Kazem Meraji, Sharun Parayil Shaji, Wolfgang Tress

**Affiliations:** 1 Institute of Computational Physics, Zürich University of Applied Science, Zurich, Switzerland; 2 Department of Chemistry, University of Zurich, Zurich, Switzerland; 3 Department of Chemistry and Applied Biosciences, ETH Zürich, Zurich, Switzerland; 4 Department of Energy, The École Polytechnique Fédérale de Lausanne, Lausanne, Switzerland; 5 Department of Mathematical Modeling and Machine Learning, University of Zurich, Zurich, Switzerland

**Keywords:** Solar cells, Solar cells

## Abstract

Perovskite solar cells (PSCs) with power-conversion efficiencies comparable to established technologies hold huge promise for becoming the future photovoltaic technology, also given their versatility, low-cost and energy-efficient fabrication processes^1^. However, PSCs are not stable under moderate reverse bias^2–4^, an unavoidable situation under real-world operation, for instance, caused by partial shading of a module or installation with PSCs connected in series. Approaches to address this issue have focused on engineering the device architecture to enhance the breakdown voltage and mitigate the detrimental effects of reverse bias^2,5,6^. Here we present a completely different approach that fully solves the reverse-bias issue. With our Memsol, we developed a new concept of a solar cell with an integrated memristor, which protects the solar cell and simultaneously works as a bypass element. The memristor is realized by area-selective deposition of an additional metal–insulator stack and shares the perovskite and electrodes with the solar-cell part. Reverse-bias and shading tests show that the Memsol remains stable and automatically toggles between a low-resistance bypass state and full-efficiency solar-cell operation, dependent on the illumination and bias conditions. We anticipate that our Memsol concept, which we demonstrated on a nine-cell string in the lab, will be implemented in large-scale modules, accelerating their commercialization and potentially making external bypass diodes unnecessary.

## Abstract (web summary)

A memristor integrated into perovskite photovoltaics aims to mitigate the issues seen with reverse-bias degradation under partial shading.

## Main

**Fig. 1:**
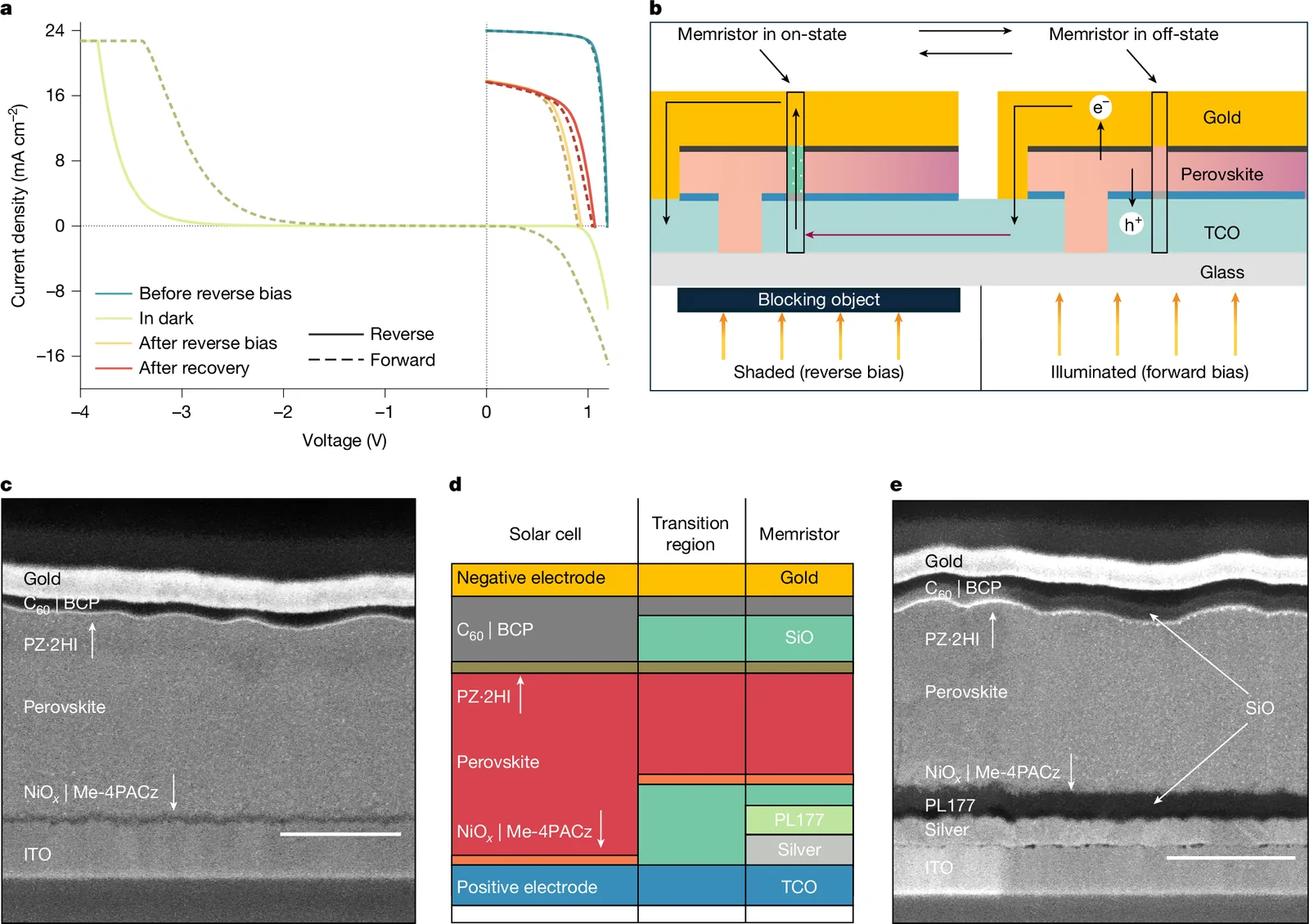
Memsol concept and realization. **a**, Current–voltage measurements of the reference cell under light, in the dark, after reverse bias and after recovery. **b**, Schematic of two Memsols (solar cell with integrated memristor) connected in series, with one experiencing reverse bias due to shading. **c**, Cross-sectional SEM image of the solar cell part of the Memsol. **d**, Schematic of the Memsol structure, showing how the memristor part is integrated within the solar cell active area. The transition region avoids that Ag is in direct contact with the solar-cell part. **e**, Cross-sectional SEM image of the memristor part. Scale bar, 400 nm (**c**,**e**).

**Extended Data Fig. 1:**
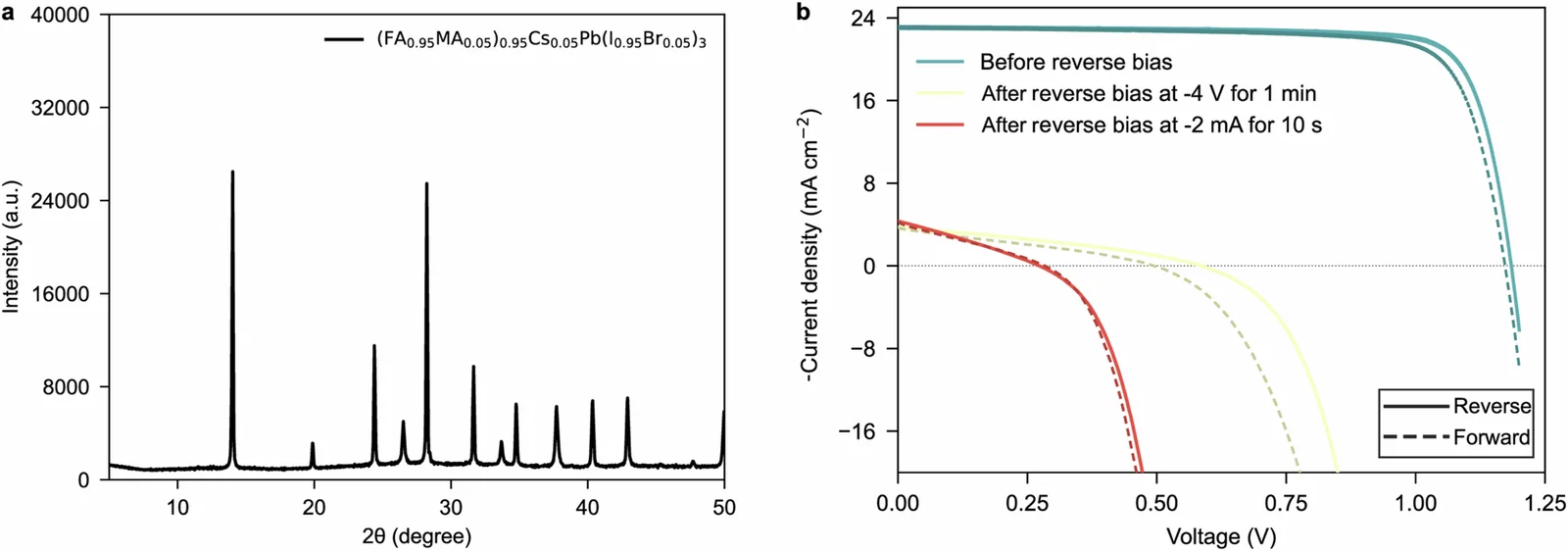
XRD and reference device reverse bias stability. (**a**) The XRD pattern of (FA_0.95_MA_0.05_)_0.95_Cs_0.05_Pb(I_0.95_Br_0.05_)_3_ perovskite. (**b**) Current-voltage curves of the reference device before reverse bias under illumination, after imposing a constant reverse current density of 24 mA/cm^2^ (−2 mA) for 10 s and after applying a reverse bias of −4 V for one minute without limiting current to 24 mA/cm^2^ demonstrating a substantial reduction in performance of the reference cell to 0.38% and 0.84 %, respectively. Solid and dashed lines indicate the voltage sweep direction.

We first demonstrate the reverse-bias issue at a state-of-the art PIN-type perovskite solar cell (PSC), hereafter referred to as the reference cell. It contains a perovskite with nominal composition (FA_0.95_MA_0.05_)_0.95_Cs_0.05_Pb(I_0.95_Br_0.05_)_3_ (Extended Data Fig. 1a; X-ray diffraction pattern) and the following stack: glass/ITO/NiO_*x*_/Me-4PACz/perovskite/piperazine dihydriodide (PZ·2HI)/C_60_/bathocuproine (BCP)/gold (Au)^7–12^. It delivers a power-conversion efficiency (PCE) of 23.2%, with a short-circuit current density (*J*_sc_) of 24.0 mA cm^−^^2^, a fill factor (FF) of 81% and an open-circuit voltage (*V*_oc_) of 1.193 V (Fig. 1a, blue curve).

Performing dark current–voltage (*I*–*V*) measurements from 1.2 V to −4 V and back, with a current limit of -2 mA (roughly corresponding to the device current under 1-sun illumination), showed a reverse-bias breakdown onset between −3 V and −4 V (Fig. 1a, green curve). Consequently, a fully shaded cell connected in series with a string of illuminated cells is exposed to a reverse bias of higher than −4 V to pass the photogenerated current. The increment in current observed in the subsequent forward scan in the dark and the decrease in efficiency to 11.5% measured under illumination (orange curve), indicate considerable damage, only with slight recovery after rest (red curve). Imposing a constant reverse current corresponding to approximately *J*_sc_ or applying a reverse bias of −4 V for 1 min without limiting the current, leads to an even more pronounced collapse in PCE (Extended Data Fig. 1b). These observations align well with previously reported behaviour, in which the performance decline has been attributed to localized hotspots, perovskite decomposition and electrode corrosion^2,5,13–15^. The underlying reasons are found in the perovskite-inherent ionic conductivity, its soft lattice and its susceptibility to interfacial reactions. Only a few reports exist in which the breakdown voltage reached –15 V (ref. ^2^) or even –20 V (ref. ^5^), facilitated by modified charge transport layers and electrodes.

## Memsol concept and structure

**Extended Data Fig. 2:**
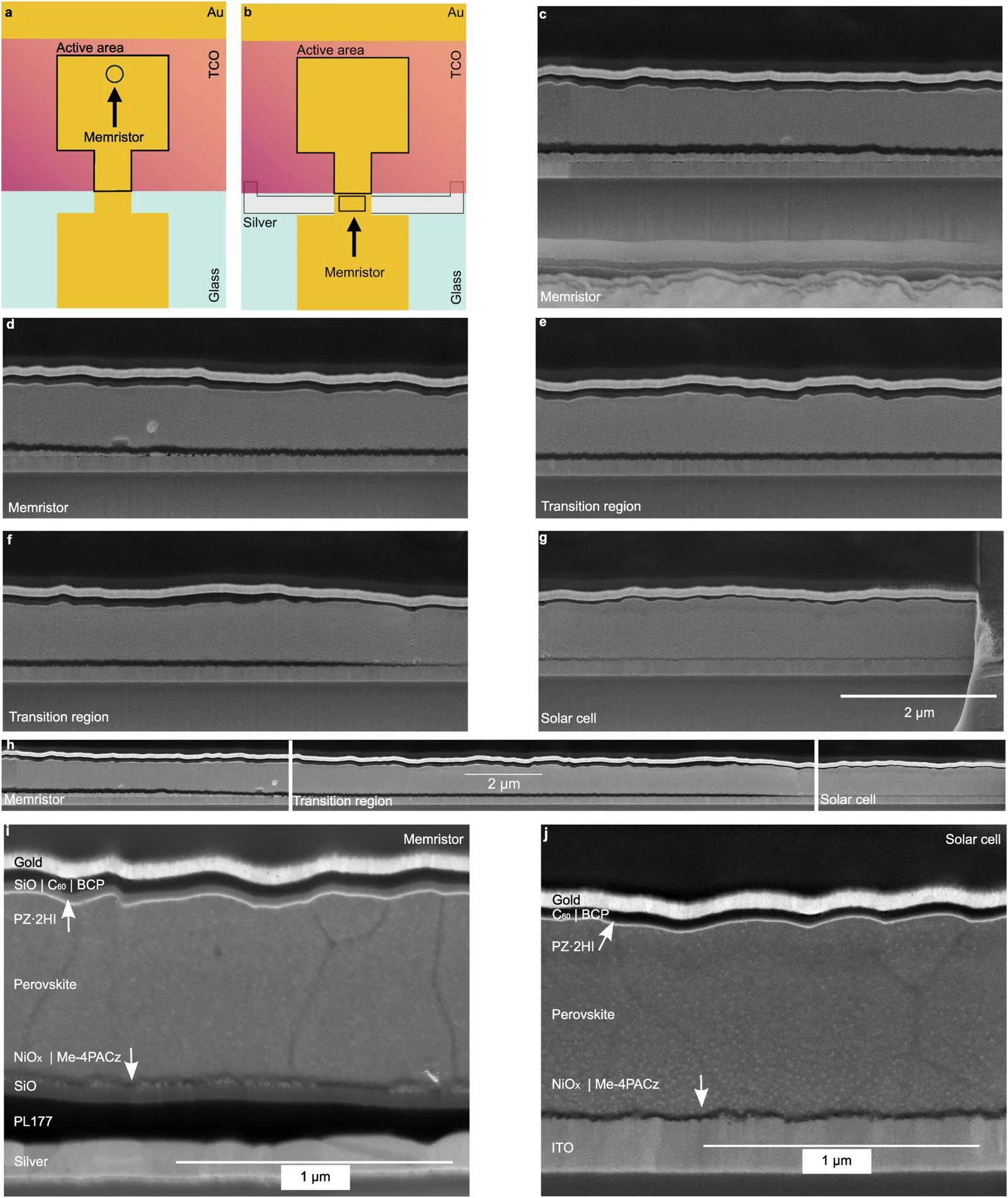
Integration possibility and complete cross-sectional SEM image of the Memsol device. Schematic top view image of the Memsol demonstrating two possibilities of memristor integration into our solar cell layout where in (**a**) the memristor is placed within the solar-cell active area and in (**b**) the memristor is placed adjacent to the solar-cell active area. Panels (**c–g**) present 5 μm-wide cross-sectional SEM windows of a Memsol device with the memristor placed within the solar cell active area (a), sequentially moving from left to right/up to down (from the memristor toward the solar cell), while panel (**h**) shows the fused image spanning the entire region. For the Memristor cross-sectional SEM image shown in the main text (Fig. 1e), higher brightness and an additional denoising filter were applied to panel (c) to enhance the visibility of the polymeric layer. To show memristor and solar cell in one SEM image, the extension of the transition region (no Ag/PL 177 but SiO) has been minimized to around 10 µm. Commonly, it is larger to avoid any direct contact of the Ag with the solar cell (see Fig. 2 in the main paper). Panels (**i** and **j**) present cross sectional SEM image of the Memsol device where memristor (i) placed adjacent to the solar cell active area (j). Compared to the cross-sectional SEM image in Fig. 1e, a clear structural difference is observed in the memristive region, where the silver bottom electrode of this configuration is deposited on the region of the substrate, where ITO has been removed.

Our new device, which we call Memsol, is designed to avoid high negative voltage that could occur from series-connected illuminated cells. It consists of a direct integration of a perovskite memristor^16–18^ in parallel with the photovoltaic element (Fig. 1b). A memristor, in general, is a non-linear resistor, the resistance of which depends on the history of the applied current^19^. In our implementation, the memristor functions as a two-state resistive switch engineered to transition to a low-resistance (few ohms) on-state under low reverse voltage and revert to a high-resistance (>gigaohm) off-state under low forward voltage. This configuration effectively shunts the current around the shaded solar cell, protecting it from high reverse bias. On re-illumination and subsequent generation of positive voltage by the solar cell, the memristor automatically switches back to the off-state (high resistance), allowing the solar cell to resume normal operation. To realize the Memsol, that is, integrate memristive functionality into the reference PSC, the memristive component can be positioned either adjacent to or within the active area of the solar cell (Extended Data Fig. 2a,b). In thin-film modules, certain zones, such as interconnect gaps or other photovoltaic inactive regions, would provide suitable ‘dead areas’, in which a memristive element can be placed. The case of direct integration into the active area (Fig. 1b) might be preferred for large-area single cells. We realized both ways, but in this study, we focus on the active-area integration based on the structure shown in Fig. 1d.

**Fig. 2:**
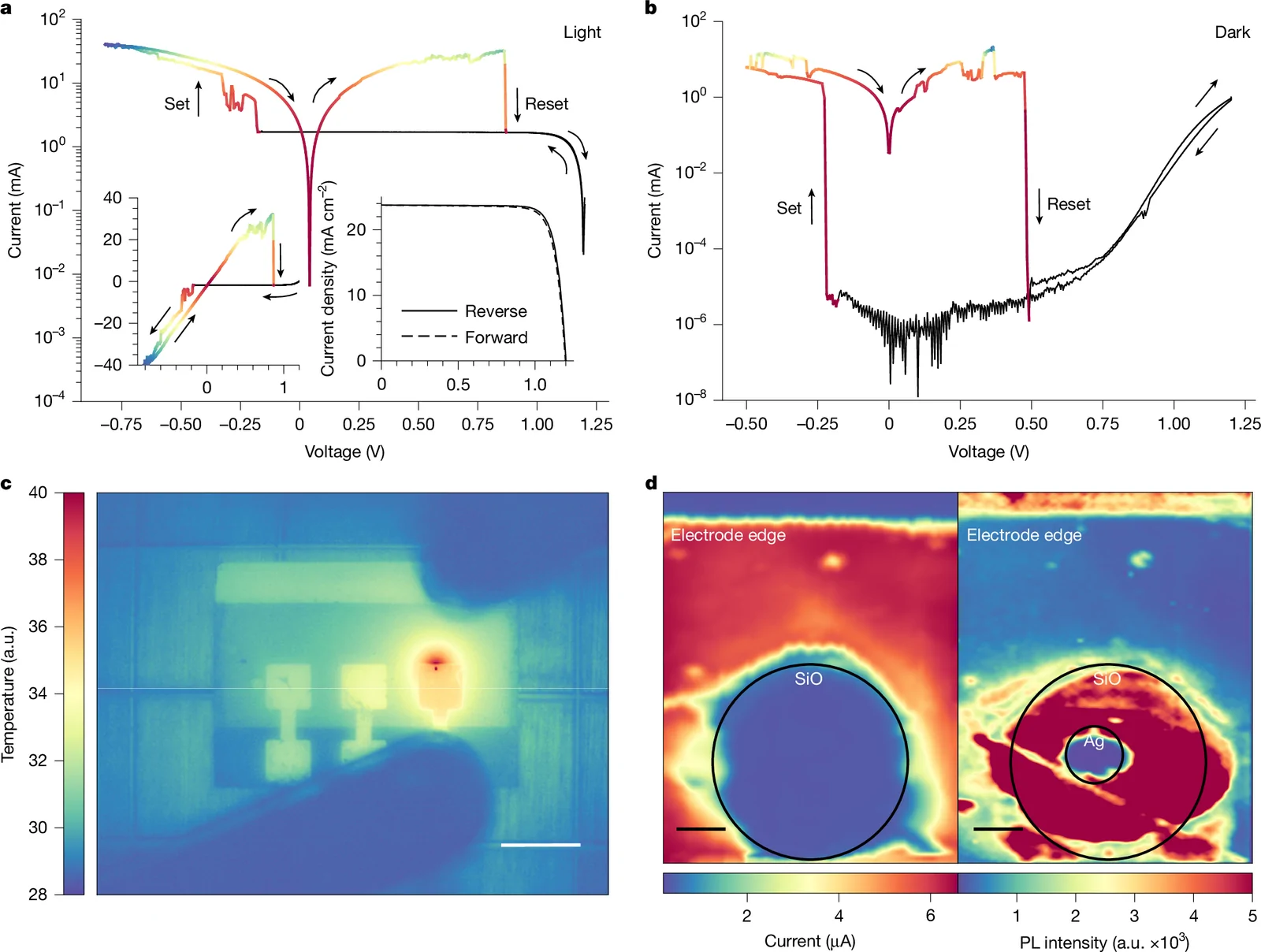
Memsol performance. **a**, *I–V* plots of a Memsol under light (left inset, same data as in the main figure on a linear current scale; right inset, measurement in the power-generating regime). **b**, *I–V* response of a Memsol in the dark. **c**, Thermography map of the Memsol under reverse bias. **d**, Current and photoluminescence (PL) maps of the Memsol (under 405 nm excitation at 0.1 V), magnified in the region in which the memristive component is integrated within the active area of the solar cell. a.u., arbitrary units. Scale bars, 5 mm (**c**); 50 μm (**d**).

Whereas the memristive component shares various layers, such as the perovskite and top electrode with the solar cell, it requires an area-selective deposition of further layers, such as a back electrode, an insulating layer (here the photoresist PL177) and auxiliary SiO layers (Methods). Passivation and charge transport layers used in the solar cell are not required but do not impede memristor functionality and are, for simplicity, coated on the entire area, yielding a memristor stack of Ag/PL177/SiO/NiO_*x*_/Me-4PACz/perovskite/PZ·2HI/SiO/C_60_/BCP/Au. Figure 1e shows the cross-sectional SEM image of this stack next to Fig. 1c, showing the solar-cell part. Both images have been cropped from the full view of the Memsol structure shown in Extended Data Fig. 2c–h.

Silver (Ag) was placed as a bottom electrode for the correct polarity of the memristor and would migrate through the layers towards the top to create a conductive channel, which can switch presumably electrochemically dependent on the applied voltage and facilitated by the surrounding perovskite^20^.

## Memsol performance metrics

**Extended Data Fig. 3:**
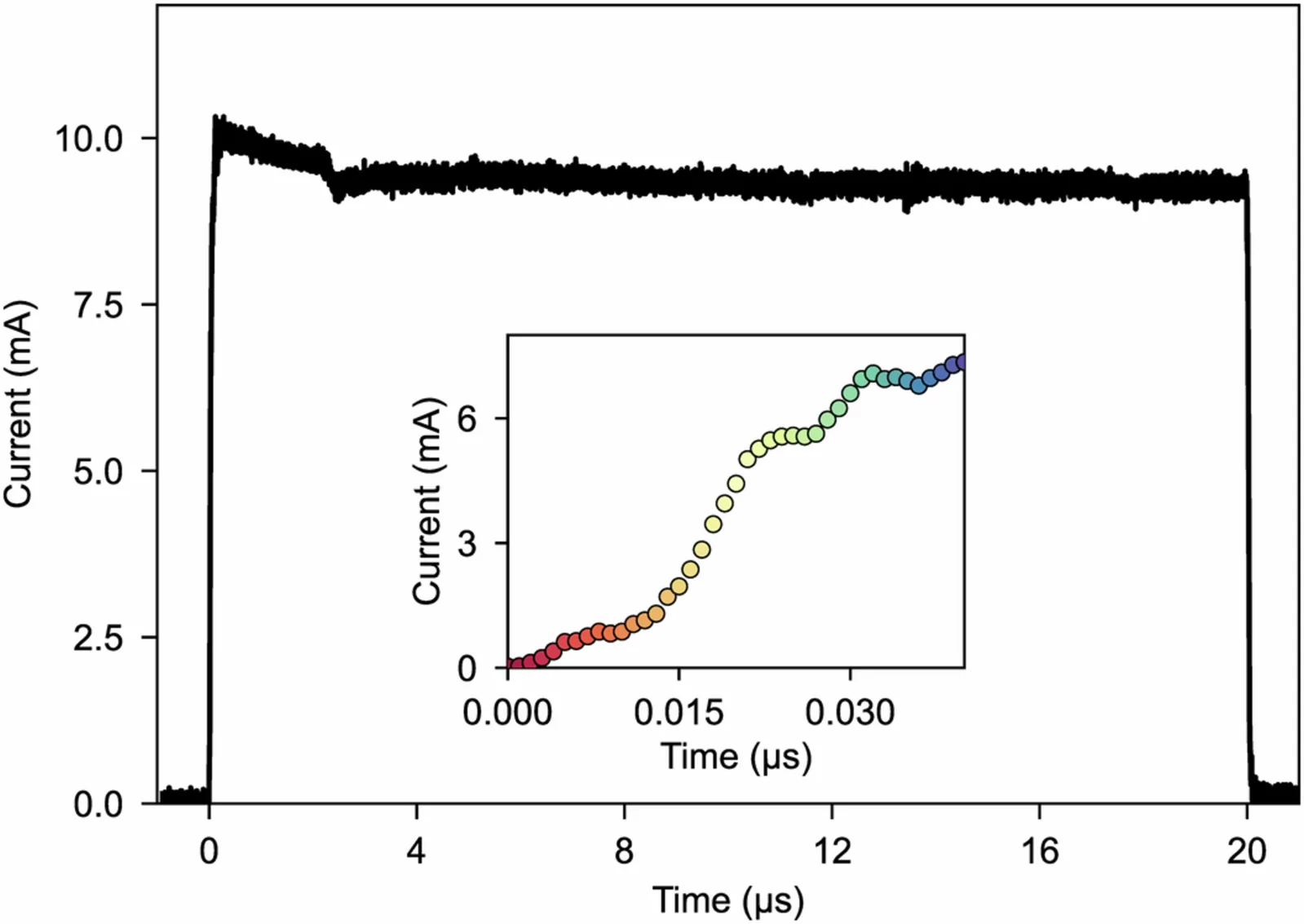
Transient switching response of the memristor. Upon application of a voltage step of −0.5 V, the device transitions from its high-resistance (off) state to its low-resistance (on) state, reaching 1 mA of current in approximately 10 ns and rising to 8 mA in less than 40 ns as the zoomed-in inset shows.

Figure 2a presents the *I–V* characteristics of the Memsol, recorded from +1.21 V to −0.75 V and back. The part of the curve coloured black resembles a typical PSC *I–V* curve, with a *V*_oc_ of 1.20 V and an *I*_sc_ of 2.1 mA. On reaching −0.18 V reverse bias, the memristor triggers to switch from the off-state to the on-state (it ‘sets’), causing a sharp current increase to about 5 mA. Continued biasing raises the on-state current to approximately 40 mA (coloured curve). The set process serves analogously to the turn-on of a bypass diode, which occurs at 0.6–0.7 V in typical silicon diodes. Moreover, the memristor switches in <50 ns to deliver roughly more than 8 mA current (Extended Data Fig. 3), nearly three orders of magnitude faster than the approximately 30 µs reverse-recovery time of a standard 1 A silicon rectifier (for example, 1N400x series).

**Extended Data Fig. 4:**
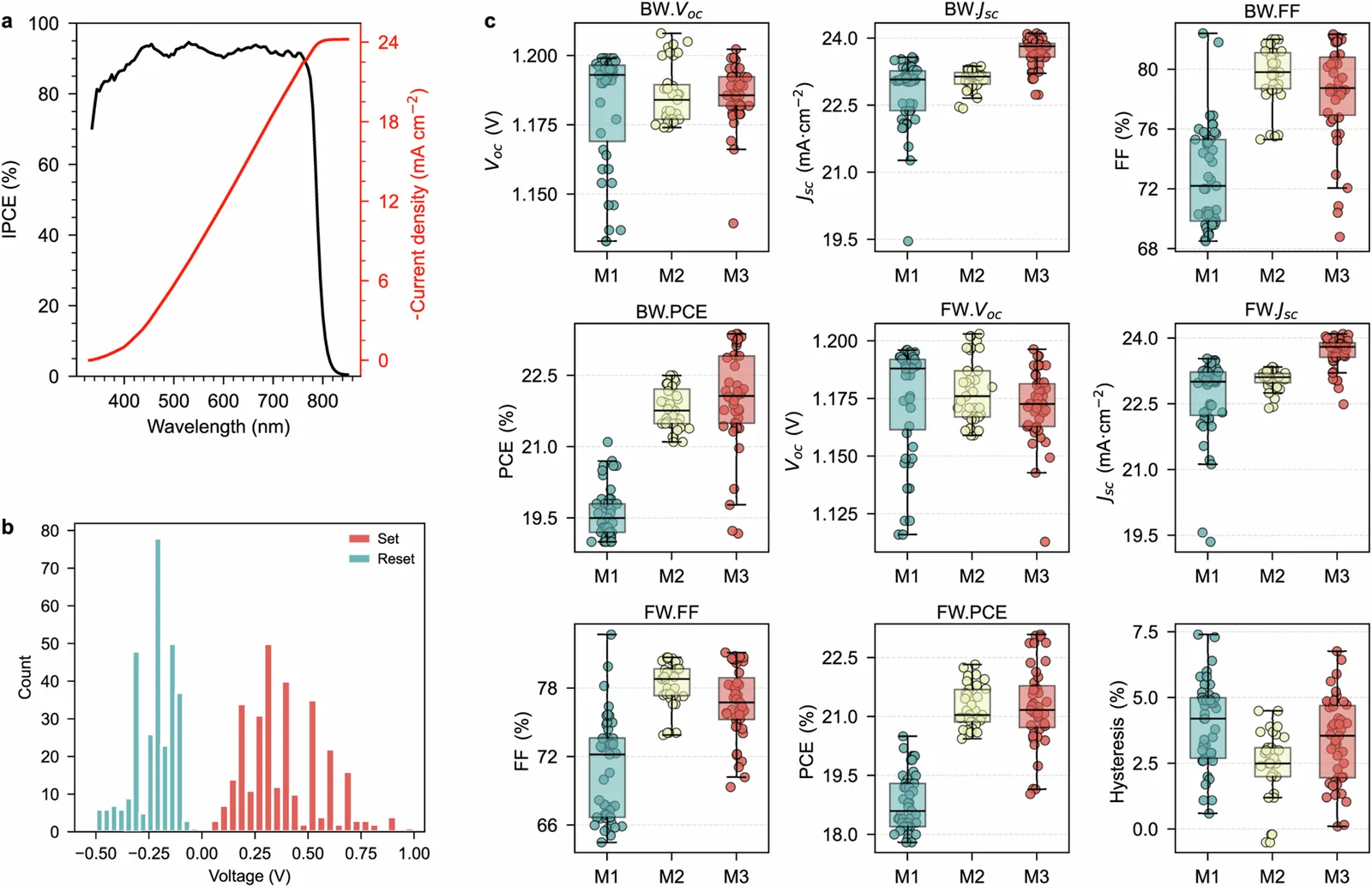
IPCE and statistical data of Memsol performance metrics. (**a**) The black curve shows the measured IPCE as a function of wavelength. The red curve represents the cumulative current density obtained by multiplying the IPCE data with the solar spectrum (photon flux) and integrating the product over the wavelength, yielding an estimated *J*_sc_ of 24 mA cm^−2^, in excellent agreement with the *J–V* measurement. (**b**) Set and reset voltage distributions obtained from 400 current–voltage measurements conducted on 10 Memsol devices under AM1.5 G illumination. (**c**) Statistics of *V*_oc_, *J*_sc_, *FF* and PCE for 125 Memsols fabricated by three selective PL-177 deposition strategies: In Method 1 (M1) the cell area is masked either with conventional adhesive tapes or by first depositing NiO_*x*_ and a SAM layer before PL-177 deposition, whereas in Method 2 (M2) a high-temperature-resistant blue dicing tape is used to minimize surface residue. Because the memristor-specific layers are thermally evaporated, the quality of the PL-177 layer largely determines device performance; the data reveal that Method 1 consistently produces lower PCE, driven by a reduced *FF* that is most plausibly linked to incomplete coverage of the hole-transport layer caused by masking-related residue. M1 and M2 were employed to integrate the memristor adjacent to the solar cell’s active region, whereas Method 3 (M3), based on lithography, was used for positioning the memristor directly within the active region of the solar cell. The hysteresis is defined as Unity minus the ratio between the PCE of forward (FW) and backward (BW) voltage sweep.

In the subsequent forward scan, the memristor remains in the on-state up to about 0.85 V, in which it resets (switches from on-state to off-state), restoring the photovoltaic *I–V* behaviour. Figure 2a (left inset) shows the same *I–V* measurement plotted on a linear scale. It impressively visualizes the desired and so-far-never-realized integrated bypass functionality of having lowest resistance (here 25 Ω) in reverse but unaffected photovoltaic operation in forward direction. The PCE deduced from the *J–V* curve is 23.4% (Fig. 2a, right inset), with a *J*_sc_ of 23.8 mA cm^−2^, a FF of 0.82 and a *V*_oc_ of 1.20 V. The *J*_sc_ matches the value of 24 mA cm^−2^ obtained from the incident-photon-to-current efficiency (IPCE; Extended Data Fig. 4a). Statistics on set and reset voltages and solar-cell parameters are shown in Extended Data Fig. 4b,c.

**Extended Data Fig. 5:**
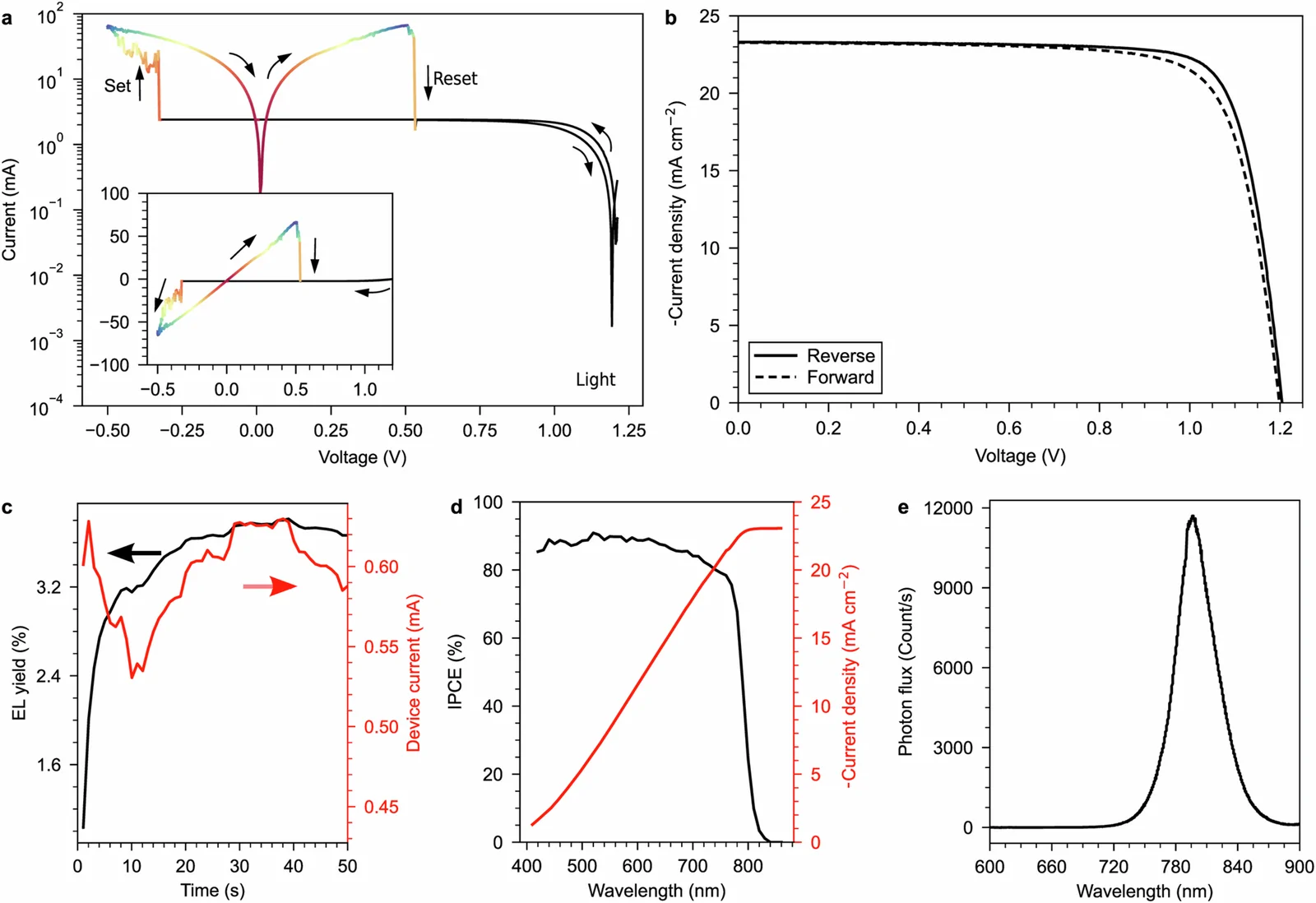
Current voltage response, Electroluminescence (EL), Incident Photon to Current Efficiency (IPCE) and Photoluminescence (PL) spectrum of a Memsol with the memristor integrated adjacent to the solar cell active region. (**a**) Current–voltage characteristics plotted on a logarithmic scale and (**b**) current-density–voltage characteristics plotted on a linear scale for a Memsol device exhibiting an exceptional *V*_oc_ of 1.205 V. This device demonstrates a PCE of 22.5%, with a *J*_sc_ of 23.3 mA/cm², a *FF* of 0.80, and a *V*_oc_ of 1.205 V. (**c**) Electroluminescence (EL) measurements performed on the Memsol shown in (a), yielding an external EL efficiency of 3.8 % at 1.21 V. (**d**) IPCE spectrum and corresponding integrated photocurrent density of the device, yielding an estimated *J*_sc_ of 23.1 mA cm^−2^, in excellent agreement with the *J–V* measurement. (**e**) Room-temperature steady-state photoluminescence (PL) spectrum of the perovskite used in the Memsol structure.

The high *V*_oc_ of the Memsol demonstrates that the implementation of the memristor does not negatively affect recombination or shunting under illumination. This is also confirmed by electroluminescence measurements performed on one of the Memsol with an exceptional *V*_oc_ of 1.205 V (*I–V* curve; Extended Data Fig. 5a,b), which yielded an external electroluminescence yield of 3.8%, measured at 1.21 V (Extended Data Fig. 5c). The deduced non-radiative voltage loss of 82 mV is in accordance with the radiative *V*_oc_ limit of 1.30 V, calculated from the IPCE spectrum (Extended Data Fig. 5d) and photoluminescence data^21^ (Extended Data Fig. 5e). Overall, the Memsol shows unchanged photovoltaic performance compared with the reference indicating that it would work similarly in further optimized PSCs.

**Extended Data Fig. 6:**
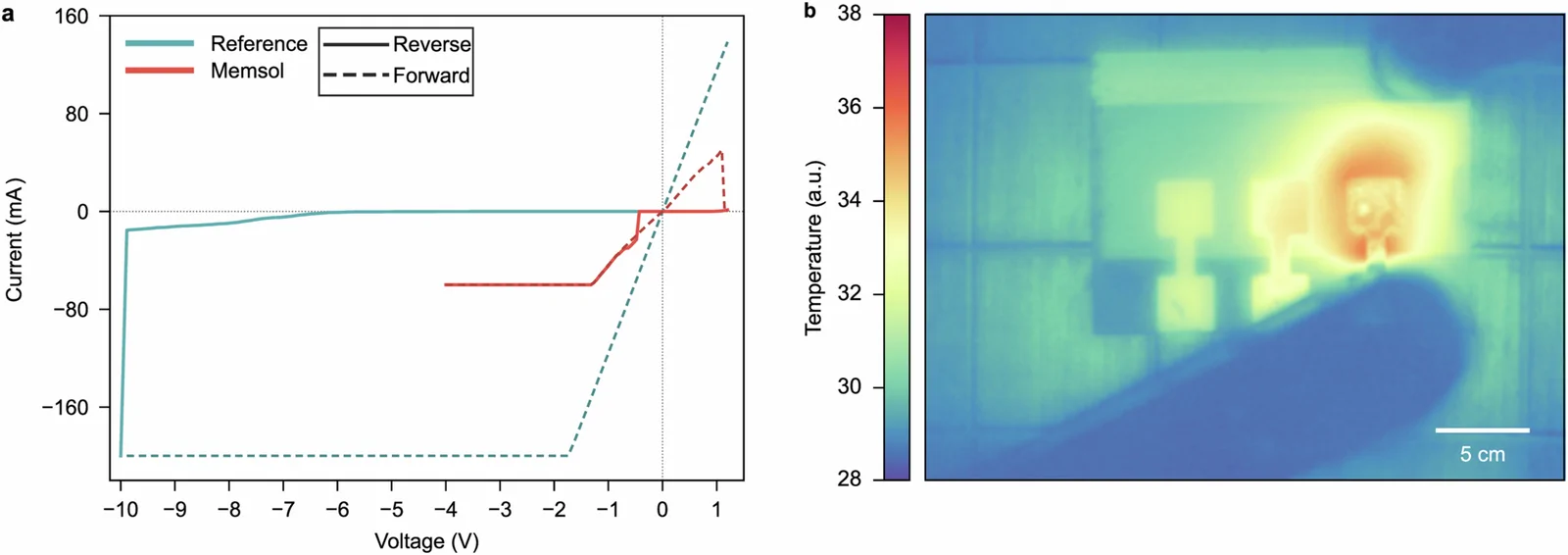
Thermography. (**a**) Current–voltage characteristics of the reference and Memsol devices during thermography imaging shown in Supplementary videos 1 and 2. (**b**) A frame from the temperature map video (Supplementary video 2) of the reference cell.

An *I–V* sweep performed in the dark (Fig. 2b) confirms that the device response is governed by the solar-cell part (black trace) until the memristor switches to its low-resistance state at negative bias (set) and again after it reverts to the high-resistance state at positive bias (reset). Although the memristor remains active (coloured trace), the dark *I–V* characteristics reflect those obtained under illumination, indicating a similarly low resistivity. Spatial localization of the associated Joule heating was probed by lock-in thermography. Figure 2c shows a frame from the temperature map video (Supplementary Video 1 and related *I–V* curve in Extended Data Fig. 6a) of the Memsol under reverse bias, revealing a confined hotspot within the active area that coincides precisely with the memristor location. The thermal signature disappears when the memristor returns to its high-resistance state under forward bias. By contrast, the reference cell exhibited uniform heating with a breakdown voltage around −6 V, accompanied by localized shunting at approximately −10 V and a pronounced thermal signature under forward bias (Supplementary Video 2 and related *I–V* curve in Extended Data Fig. 6a, a frame of the temperature map Extended Data Fig. 6b).

**Extended Data Fig. 7:**
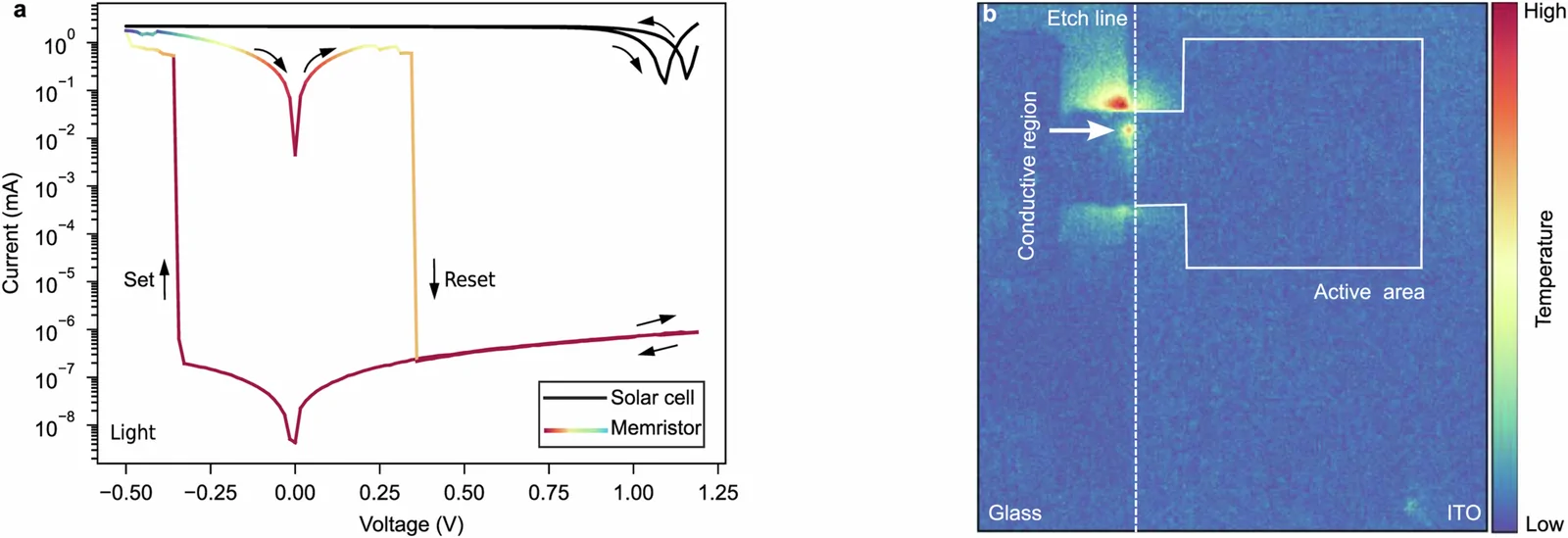
Current-voltage response and thermography map of the Memsol device with memristor placed adjacent to the solar cell active area. (**a**) To evaluate potential power loss arising from non-zero off-current in the memristor, simultaneous *I–V* measurements under illumination were performed using two independent ammeters to separately record memristor and solar cell currents. The off-state memristor current remained seven orders of magnitude below the solar cell photocurrent, corresponding to a parallel resistance of 1.5 × 10^9^ Ω. This resistance exceeds the solar cell’s shunt resistance (~5 × 10^7^ Ω, estimated from the dark curve in Fig. 2b) by approximately 30-fold, indicating that memristor leakage contributes negligibly to overall power loss. (**b**) Thermography map of the Memsol recorded under reverse bias, revealing the localized heating at the memristive component positioned adjacent to the solar cell’s active region.

A simultaneous but separate measurement of memristor and solar-cell current showed that the shunt resistance remains dominated by the solar-cell part, rendering the off-state resistance fully negligible, even under low-intensity illumination (Extended Data Fig. 7).

Thus far, it has been demonstrated that the incorporation of the memristive element neither enhances carrier recombination nor induces shunting under photovoltaic operation. However, the potential reduction in photocurrent arising from the decrease in the effective active area due to the incorporated memristive component has not yet been addressed. Figure 2d shows the current and simultaneously recorded photoluminescence intensity map, measured at 0.1 V under illumination. The current map (Fig. 2d, left) shows that within the SiO area of about 200 µm in diameter, the photocurrent vanishes, whereas it quickly approaches its full value further away. The corresponding photoluminescence map in Fig. 2d (right) shows a roughly circular area of about 50 µm in diameter (dark blue) with negligible photoluminescence emission, corresponding to the silver pad that blocks the excitation light. In the transition region (Fig. 1d and Extended Data Fig. 2), in which the perovskite layer can be optically excited (no Ag) but is electrically insulated by the SiO layers, photoluminescence is bright. The photoactive area is identified by the photoluminescence intensity dropping due to the extraction of the photogenerated charge carriers. Considering an active solar cell area of 9 mm^2^, the footprint of the memristive component (0.03 mm^2^) is below 1% of the total area, which decreases further when increasing the device area (see below).

## Reverse bias to open-circuit cycling

Standard reverse-bias qualification of photovoltaic modules is prescribed by IEC 61215−2:2016, which subjects devices to a hotspot endurance test under constant reverse bias to mimic partial shading and assess thermal resilience^6,22^. This protocol is not directly applicable to the Memsol architecture, whose integrated memristor suppresses large reverse potentials by switching into a low-resistance state. For the Memsol, it is sufficient to verify that a shaded device reliably conducts the photocurrent delivered by the illuminated part of the string or module.

**Fig. 3:**
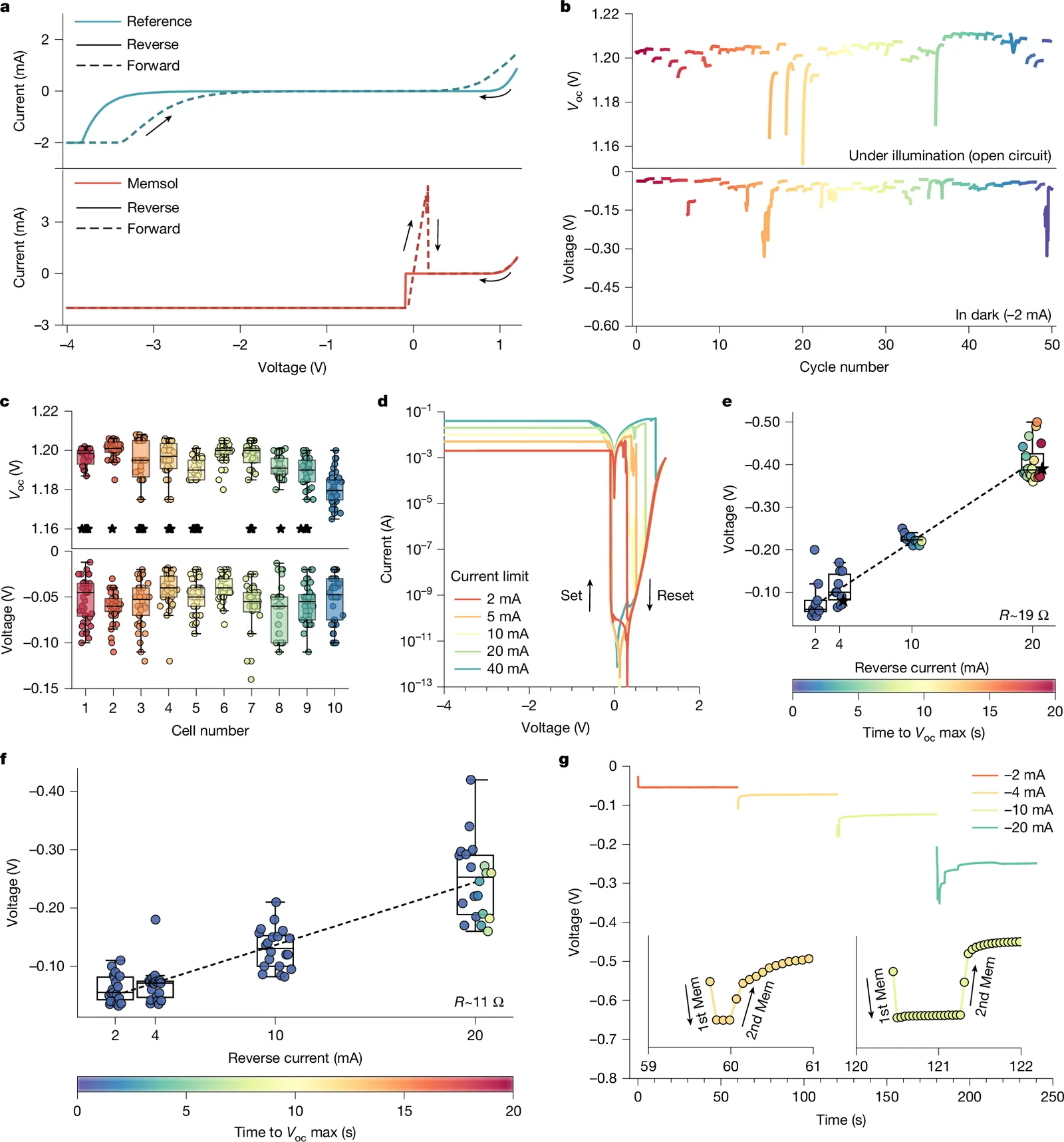
Cycling, limitations and double-memristor Memsol. **a**, *I*–*V* curve of reference (replotted from Fig. 1a) and Memsol in the dark with a current limit set to −2 mA. **b**, Fifty cycles of Memsol at −2 mA in the dark (60 s) and open circuit under light (6 s). For each cycle, the bottom panel shows the resulting reverse voltage for the dark period and the top panel the subsequent open-circuit voltage *V*_oc_. **c**, Memsol set voltage and *V*_oc_ under illumination collected over 400 cycles in total, performed on 10 different Memsol devices with 40 cycles each. Black stars indicate instances in which the device did not successfully reach its expected *V*_oc_. Boxes show the interquartile range (25th–75th percentile) with the median as a horizontal line; whiskers extend to the most extreme values within 1.5× the interquartile range and caps mark whisker endpoints. Circles show individual data points. **d**, Dark *I–V* characteristics of the Memsol under different current limits. **e**, Operation voltage and time to maximum *V*_oc_ for the Memsol during dark (60 s)/light (30 s) cycling under various reverse currents. Stars indicate instances in which the full *V*_oc_ has not been reached. **f**, Same as **e**, but for Memsols incorporating two memristors. **g**, Voltage compared with time for the double-memristor Memsol on gradually increasing the reverse current; the insets are magnified images showing the delayed decrease in the absolute set voltage as a result of the turn-on of the second memristor (Mem).

**Extended Data Fig. 8:**
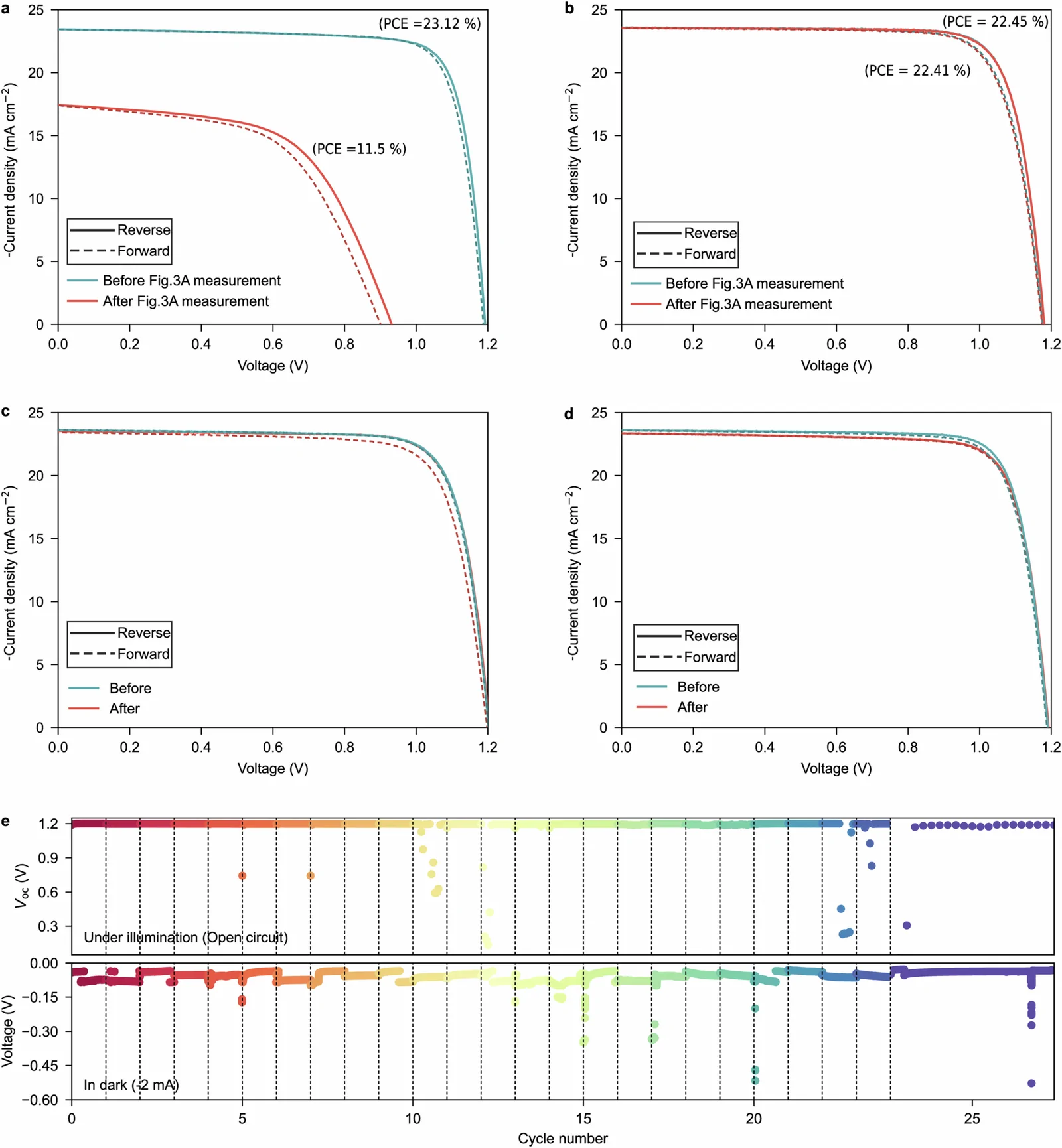
Reference and Memsol device performance variation under reverse bias and cycling. The current density–voltage (*J-V*) curves of the (**a**) reference and (**b**) Memsol devices, measured before and after the experiment shown in Fig. 3a. (**c**) *J-V* curves of the Memsol device used in Fig. 3b, before and after the full cycling test. (**d**) *J-V* curves of the Memsol device used in Fig. 3e, before and after the full cycling test. (**e**) Same as the measurement in Fig. 3b, but for longer cycles (24 cycles with 1 h at −2 mA in the dark, 20 s under light, followed by 24 h at −2 mA and 20 s under light). Illumination cycles 10, 12, and 22 demonstrate a failure mode where the memristor turned on for a few seconds during illumination, leading to a temporary reduction in *V*_oc_. The resulting power loss over the shown cycles would remain below 3%, crudely estimated by assuming proportionality to *V*_oc_ and calculated by time-integrating *V*_oc_ and normalizing to a reference open-circuit voltage of 1.2 V.

To assess this behaviour and remain within the current boundaries of serially connected solar cells, we perform a voltage sweep in the dark from forward voltage to −4 V while limiting the current to −2 mA. As shown in Fig. 3a, the memristor shows the expected behaviour. It turns on at approximately −0.1 V and in the subsequent forward scan turns off at around 0.15 V after a linear range and followed by the typical solar-cell behaviour up to 1.2 V. For negative voltages <−0.1 V, the current limit is reached, which effectively pins the Memsol voltage at about −0.1 V independent of the applied negative voltage, meaning for the module, independent of the number of serially connected solar cells. By contrast, the reference device reaches the current limit at higher negative voltages, causing uncontrolled heating and breakdown. The resulting performance loss of the reference compared with the Memsol is evident from the *J–V* curves shown in Extended Data Fig. 8a,b.

In contrast to the measurement conditions so far, for which actively current and voltage are applied (Figs. 2 and 3a), under real-world module operation, the memristor is expected to revert autonomously to the high-resistance (off) state under the self-generated forward bias due to illumination. At open circuit, the Memsol voltage should therefore match the *V*_oc_ of the underlying solar cell.

This behaviour was evaluated by subjecting the Memsol to consecutive dark (60 s) and light (6 s) cycles, in which –2 mA was applied in the dark and the Memsol was kept at open circuit during the light period. As shown in Fig. 3b (bottom), the memristor switches on promptly under reverse current and its voltage stabilizes at around −0.05 V, yielding a 25 Ω resistance. When the Memsol is subsequently illuminated (Fig. 3b, top), the memristor resets mainly within the first second, and the voltage of the Memsol quickly approaches a *V*_oc_ of around 1.2 V for each cycle. This robust and reversible switching protects the Memsol from degradation, as evident from the *J–V* curves before and after the 50 cycles in Extended Data Fig. 8c. The low voltage of around −0.05 V implies a power loss in the range of 5% of the power output of a single illuminated cell, when operated at maximum power point of about 1 V, whereas in the common approach of a high-breakdown voltage, the full power of the illuminated cells would be lost.

The reliability is further confirmed by collecting data from 40 dark–light cycles of 10 different devices (in total 400 cycles) shown in Fig. 3c. In a few instances, marked with a star, the memristive component either failed to switch off or did not switch off completely, resulting in a Memsol *V*_oc_ notably below 1.2 V. Although this failure mode does not compromise the protection function, it is undesired because it leads to temporary power losses. A further test with increased periods as detailed in Extended Data Fig. 8d,e shows evidence for suppressed incomplete memristor turn-off events.

**Extended Data Fig. 9:**
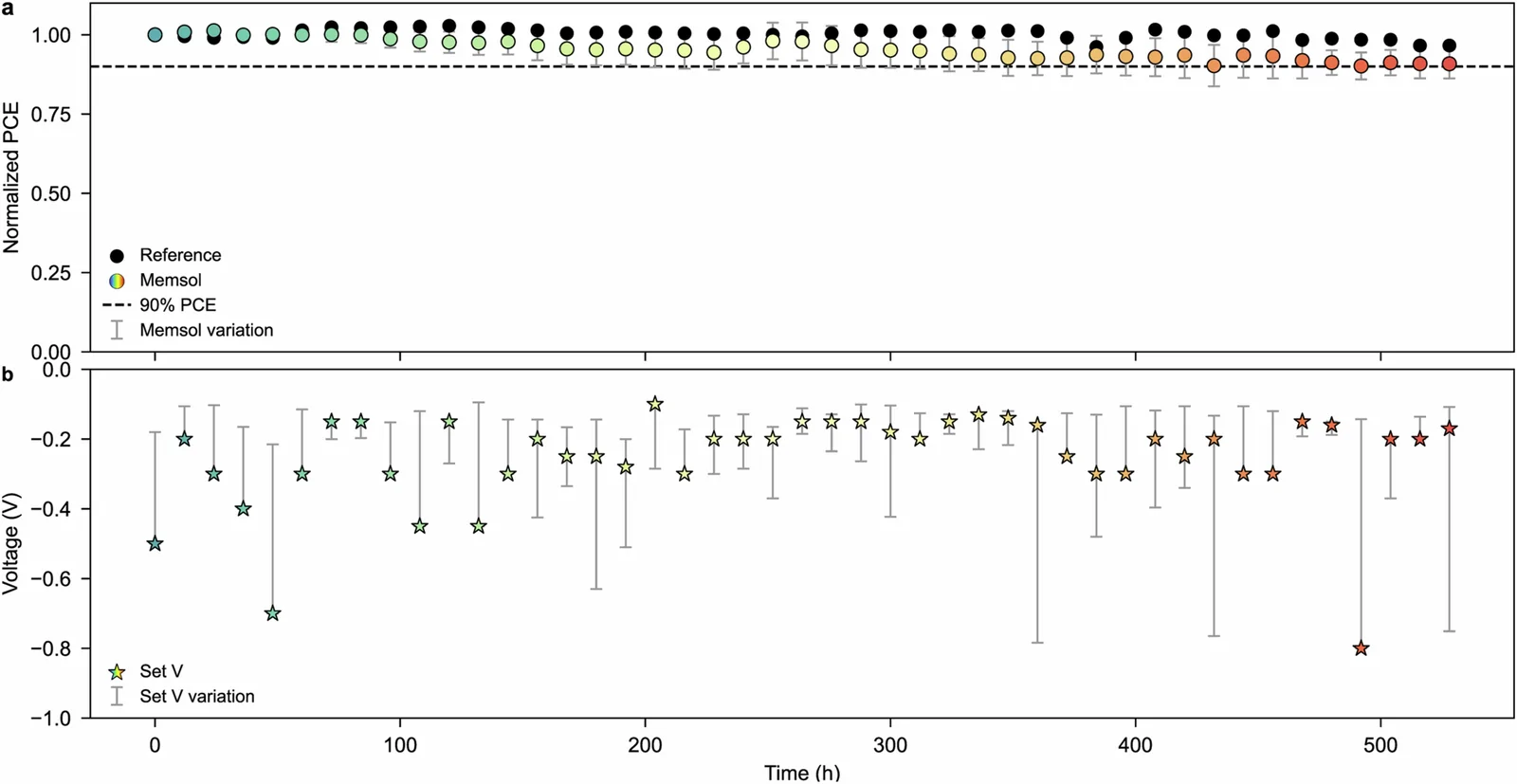
Thermal Stability at 65 °C. (**a**) Evolution of device efficiency for reference and five Memsol devices during storage in the dark under N^2^ at 65 °C, periodically recorded through *I–V* measurements. (**b**) The set voltage of the Memsol as determined from the *I–V* curve in reverse bias. Error bars indicate variability across repeated measurements at each time point. In panel (a), symmetric error bars represent half of the 10–90th percentile range of replicate Memsol PCE traces. In panel (b), asymmetric error bars indicate the 10th and 90th percentiles of replicate set-voltage measurements relative to the main trace.

Measurements under elevated temperature (Extended Data Fig. 9a) show that the memristor works reliably and did not cause a faster degradation of solar-cell performance.

## Limitations and scalability

So far, scalability with reverse voltage, which would relate to the number of series-connected cells in a module, has been shown. However, the maximum current was limited to 2 mA specific to the used active area of the lab-scale test cell. In a real module, the area can reach hundreds of cm^2^. Accordingly, we tested the Memsol under different current limits.

The dark *I–V* characteristics in Fig. 3d show that the set remains at −0.1 V, independent of the current limit, whereas the reverse bias required to reach the current limit increases from below −0.1 V to about −0.7 V. Figure 3e shows a linear relationship between operating voltage and reverse currents as expected from the resistive behaviour of the Memsol. Figure 3d and also the colour-coded time until *V*_oc_ is reached in Fig. 3e show an important limitation of the Memsol.

With increased current, the reset voltage increases, which also explains the different reset voltages observed in Figs. 2a and 3a. In operation, the memristor needs to autonomously turn off, triggered by the self-generated forward voltage of the solar cell, which is limited by the *V*_oc_. Thus, the *V*_oc_ defines an upper limit for the reset voltage and therefore the allowable reverse current, which should remain below approximately −40 mA.

To allow for higher currents and thus realistic solar-cell areas, more than one memristive component has to be integrated. We tested its general feasibility by fabricating a Memsol with two memristive components within the active area and repeated the experiment from Fig. 3e. Figure 3f shows that the operation voltage for the higher currents is roughly half and the *V*_oc_ is reached faster, as expected when two memristive components are operated in parallel. The current transients, shown in Fig. 3g, show the successful turn-on of the second memristive component, which is slightly delayed as the voltage drop indicates. This delay might cause local overheating, and synchronous switching on the module scale is still to be demonstrated, in which approximately one or two memristive elements are required per cm^2^. In a thin-film module with one electrode being a transparent conductive oxide (TCO), the memristors should be homogeneously distributed to avoid high lateral currents and thus large voltage drops on the TCO.

## Module integration and MPP measurement

**Extended Data Fig. 10:**
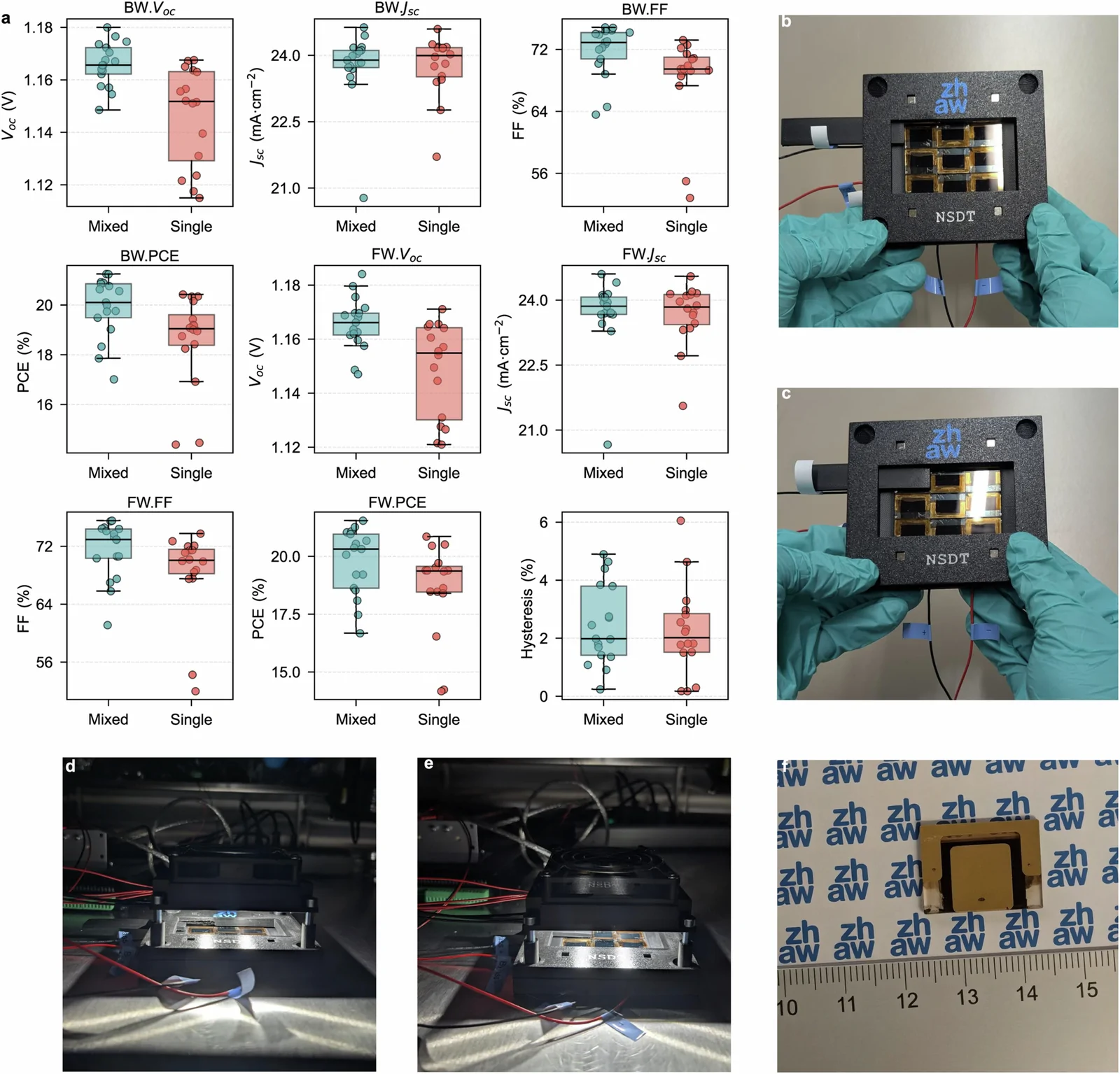
Nine-solar-cell string and MPP measurement. (**a**) Statistical data from solar cells with enlarged active areas show that devices incorporating mixed SAMs (Me-4PACz: 2PACz) exhibit higher efficiencies, primarily due to enhanced *FF* and *V*_oc_. These improvements likely arise from the superior surface coverage achieved by mixed SAMs compared to single-SAM (Me-4PACz). (**b**) Photograph of the minimodule loaded with nine enlarged cells. A small black shutter positioned at the top-left of the cap is used to shade one Memsol. (**c**) Photograph of the minimodule with the shaded Memsol. (**d** + **e**) Photograph of the minimodule placed inside the glovebox, with the LEDs and their cooling fan positioned above, shown with (**d**) the shutter closed and with (**e**) the shutter open. (**f**) Photograph of the enlarged Memsol device used for the MPP measurements, with an active area of 0.65 cm².

To show a first lab-scale proof-of-concept module integration, a nine-solar-cell string was assembled and coupled to a maximum-power-point (MPP) tracker. A photograph of this mini-module is shown in Extended Data Fig. 10, together with performance metrics of multiple devices.

**Fig. 4:**
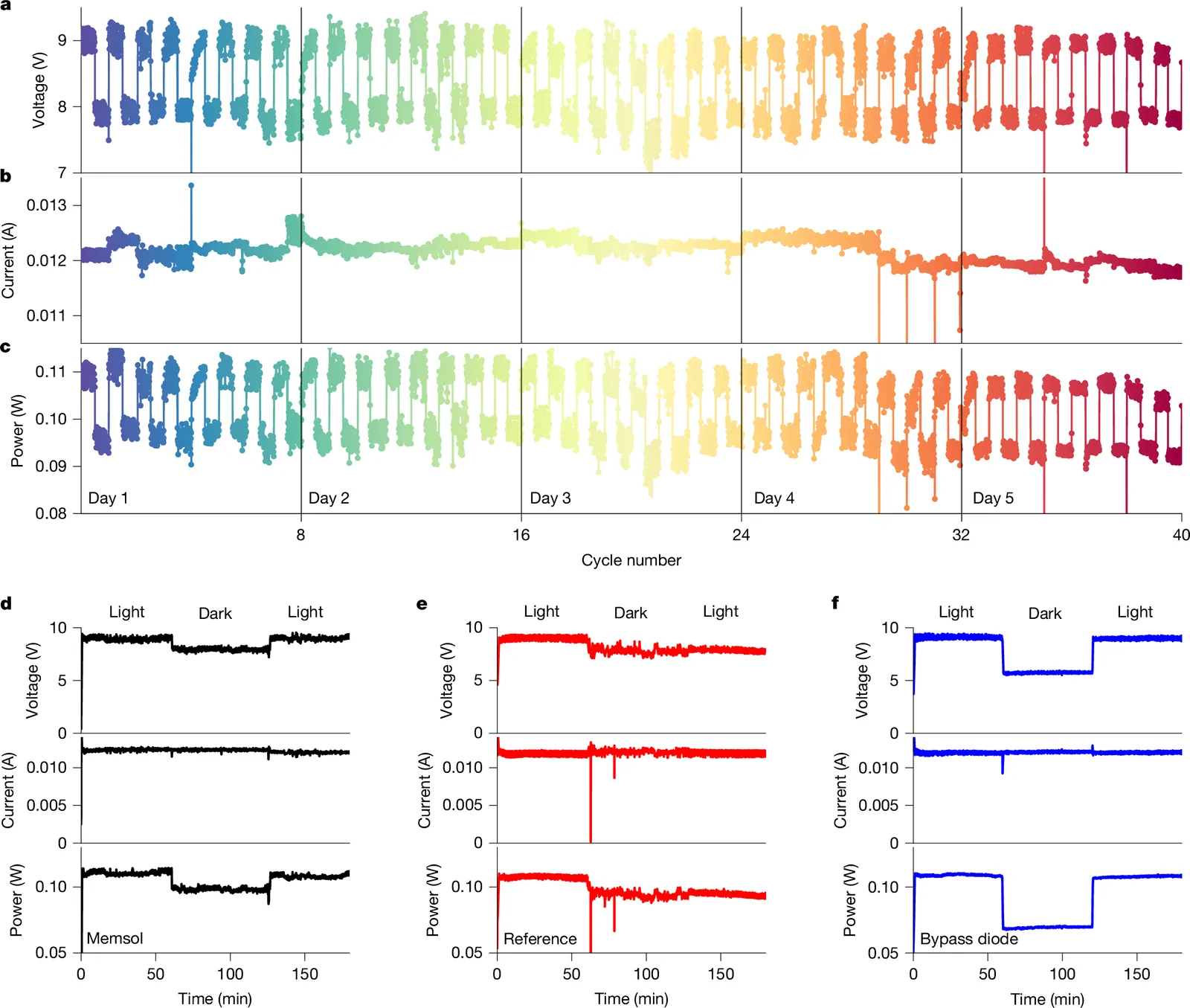
Partial shading measurement of a solar-cell string at MPP. **a**–**c**, Voltage, current and power compared with time for nine solar cells (area 0.65 cm^2^) connected in series under constant 1-sun illumination, in which one Memsol device was periodically shaded (40 cycles in total). **d**–**f**, One light–dark–light sequence of the string containing one shaded Memsol device (**d**), unprotected reference cell (**e**) and reference cell (**f**) within a three-cell sub-string protected by a bypass diode.

The string was kept under continuous illumination, with one solar cell intentionally shaded approximately every hour. After 16 h of the illumination phase, the entire string was placed in the dark for 8 h, during which MPP tracking was paused, and measurements resumed the following day. The corresponding voltage, current and power traces of 40 consecutive dark–light cycles are shown in Fig. 4a–c. When a single Memsol was shaded (Fig. 4a), the maximum power point voltage (*V*_mpp_) dropped from 9 V to approximately 8 V, whereas the total current remained stable at around 12 mA (Fig. 4b). Consequently, the output power decreased by about 14 mW from its initial 110 mW (Fig. 4c), indicating effective current bypass of the shaded cell through automatic memristive switching. On re-illumination, *V*_mpp_ recovered to about 9 V without any instance of failure, and the power returned reliably and reproducibly to its initial value of approximately 110 mW.

One light–dark–light sequence is shown in more detail in Fig. 4d to be compared with the case of an unprotected reference cell (Fig. 4e). Once the reference cell is shaded, the current and thus power drop to 0 shortly, before breakdown happens and the current recovers. In contrast to the Memsol, this breakdown damaged the cell, which behaves as a permanent shunt, as obvious from the fact that the string does not return to its initial power after re-illuminating the cell. To also compare with the conventional case of a protection with a bypass diode, we selected the diode 1N5819 and connected it in parallel to three cells to mimic the practical application case, in which multiple cells are protected with one bypass diode as additional external component. The recovery of voltage and power under re-illumination (Fig. 4f) proves the protection functionality. However, it comes with a higher penalty, because all the three bypassed solar cells are inactive despite only one being shaded. Moreover, around 0.4 V are lost in the bypass diode compared with <0.1 V in the Memsol. Overall, the total power loss for the bypass diode scenario would be 0.04 W compared with 0.01 W for the Memsol. This example demonstrates the advantage of the Memsol having a low-resistance bypass functionality integrated in each solar cell. As a next step towards real-world application, the Memsol concept must be scaled and, in particular, its long-term stability tested in larger modules and photovoltaic systems.

## Methods

### Materials

Lead (II) Iodide (99.99%, trace metals basis), [4-(3,6-dimethoxy-9*H*-carbazol-9-yl)butyl]phosphonic acid (MeO-4PACz) (≥98.0%(T)(HPLC)), [4-(3,6-dimethyl-9H-carbazol-9-yl)butyl]phosphonic acid (Me-4PACz), [2-(9H-carbazol-9-yl)ethyl]phosphonic acid (2PACz), fullerene C_60_ (purified by sublimation) (>99.5%(HPLC)), bathocuproine (purified by sublimation) (>99.0%(T)(HPLC)) and piperazine dihydriodide (>98.0%(T)(N)) were purchased from TCI. Formamidinium iodide (≥99%, anhydrous), methylammonium iodide (≥99%, anhydrous), lead (II) bromide, methylammonium bromide (≥99%, anhydrous), methylammonium chloride (≥99%, anhydrous), caesium iodide (AnhydroBeads, 99.999% trace metals basis, perovskite grade), dimethyl sulfoxide (anhydrous, ≥99.9%), *N*,*N*-dimethylformamide (anhydrous, ≥99.8%), chlorobenzene (anhydrous, ≥99.8%), ethanol (99.5%, anhydrous), methanol (99.5%, anhydrous), isopropanol (IPA) (99.5%, anhydrous) and anisole (99.7%, anhydrous) were purchased from Sigma-Aldrich. PL177 was purchased from MicroChemicals. Silver and silicon monoxide (SiO) evaporation sources were obtained from Kurt J. Lesker. NiO_*x*_ was purchased from Advanced Election Technology Co., Ltd. Fluorine-doped tin oxide (FTO) and indium tin oxide (ITO) substrates were purchased from Suzhou Shang Yang Solar Technology Co., Ltd.

### Memsol fabrication

#### Substrate cleaning and preparation

 FTO and ITO substrates were sequentially cleaned in ultrasonic baths of tap water, deionized water, acetone, isopropanol and ethanol (20 min each). Substrates were dried under a nitrogen (N_2_) stream. FTO was then annealed at 500 °C for 1 h; ITO received no thermal treatment. All substrates were treated by ultraviolet ozone (UVO) for 10 min before metallization.

#### Silver pads and mask definition

Memristor performance metrics are largely influenced by the type of electrode used, which can be either active or reactive: an active electrode (for example, Ag) yields higher on–off current ratio, faster switching speed and lower switching voltages^23^. As these are essential for applying the memristor as a bypass element, Ag was used. Ag contact pads were thermally evaporated through a shadow mask with aperture size of 50 μm onto the etched TCO regions and solar cell active region, for memristive component implementation adjacent and within the solar active region, respectively.

#### PL177 preparation and coating

Although a simple metal/perovskite/metal configuration has been shown to exhibit resistive switching behaviour^24^, the insertion of an insulating layer between the perovskite and electrodes both suppresses parasitic electrochemical reactions and lowers off-state currents, thereby improving device stability and on–off current ratios^25,26^. As an insulating layer, PL177 positive photoresist was chosen for its compatibility with standard lithographic processing, enabling precise placement of the memristor within the active area of the cell. PL177 positive resist was prepared by mixing 0.5 ml PL177 with 1.0 ml anisole (ambient conditions). The solution was subjected to spin-coating at 5,000 rpm for 30 s with a 1,000 rpm s^−1^ acceleration to cover the silver pads. (Resulting thickness is expected to be of the order of a few times 10 nm under these conditions.) A soft bake was performed at 100 °C for 10 s, then the substrates were cooled to room temperature.

#### Bottom-side exposure using silver as the mask

For patterning, the silver pads served as the opaque mask, thus not requiring alignment of any extra lithography mask. Bottom-side exposure was carried out at 365 nm using a UV-12S LED exposure unit with a small sample-to-window gap. The exposure was 60 s to deliver an approximate dose of 2 mJ cm^−2^. No post-exposure bake was used.

#### Development

The illuminated resist was developed in AZ 340 developer diluted 1:5 with deionized water. Substrates were gently agitated during an 8 s immersion, rinsed with deionized water, and dried under N_2_. This process removed resist everywhere, except above the silver pads, leaving PL177 selectively on the metal.

#### Alternative selective-deposition methods

For configurations in which the memristor was placed adjacent to—rather than within—the photovoltaic active area, a deliberate gap was introduced during device fabrication between the silver bottom electrode and the ITO layer to electrically isolate the memristor. The memristor was positioned within the ITO-etched region of the substrate. To achieve this layout, two masking strategies were used before PL177 coating:

- Method 1 (M1): masking the cell area with conventional adhesive tape or by first depositing NiO_*x*_ and a self-assembled monolayer (SAM), then coating PL177.
- Method 2 (M2): masking with a high-temperature-resistant blue dicing tape to minimize residues. Because all memristor layers are thermally evaporated, device performance was sensitive to PL177 quality; M1 consistently yielded lower PCE (primarily by reduced FF), plausibly because of masking-related residue causing incomplete HTL coverage (Extended Data Fig. 4c).

M1 and M2 were used for integration adjacent to the active region, whereas the lithographic route above (method 3) enabled placement directly within the active region.

#### Dielectric protection and pre-HTL treatments

To improve PL177 poor wetting and to protect it from partial dissolution during perovskite deposition, a thin SiO film was evaporated atop PL177 before perovskite deposition. Silicon monoxide (SiO) was thermally evaporated onto the PL177 surface through a shadow mask at 0.5 Å s^−1^, with a final thickness of 60 nm. A subsequent UVO clean (20 min) was performed.

#### Hole-selective interface and perovskite

A NiO_*x*_ nanoparticle dispersion (10 mg ml^−1^ in deionized water) was subjected to spin-coating at 1,500 rpm for 30 s and annealed at 150 °C for 10 min (ambient air). An SAM solution (0.5 mg ml^−1^ in MeOH) of Me-4PACz or a mixed SAM of Me-4PAz: 2PACz (v:v = 2:1) was subjected to spin-coating at 3,000 rpm for 30 s and annealed at 100 °C for 10 min. The perovskite precursor (1.5 M) was prepared in DMF/DMSO = 4:1 with stoichiometry Cs_0.05_(FA_0.95_MA_0.05_)_0.95_Pb(I_0.95_Br_0.05_)_3_, with 5 mol% excess PbI_2_ and 25 mol% MACl. After overnight stirring, 45 µl was subjected to spin-coating using a two-step program (2,500 rpm, 10 s; then 5,000 rpm, 40 s) with chlorobenzene antisolvent dropped 30 s before the end of the second step. Films were annealed at 100 °C for 30 min.

#### Surface passivation

A piperazine dihydriodide solution (1 mg ml^−1^ in IPA, stirred overnight) was dynamically subjected to spin-coating on the perovskite at 5,000 rpm for 25 s and annealed at 100 °C for 10 min. After cooling, the film was rinsed with IPA under the same spin conditions (5,000 rpm, 25 s) and re-annealed at 100 °C for 10 min.

#### Top-side dielectric, electron transport and electrode

A second conformal SiO layer was subjected to selective thermal evaporation by PVD over the perovskite/passivation stack above the silver pads at 0.5 Å s^−1^ (thickness set by time) before Au evaporation to inhibit deleterious electrode–perovskite interfacial reactions and to reinforce long-term stability. C_60_ and bathocuproine (BCP) were then thermally evaporated by PVD at 0.1 Å s^−1^ and 0.2 Å s^−1^, respectively. Finally, a 70 nm Au electrode was thermally evaporated defining the active area of the solar cell.

### Current–voltage measurements

All *I*–*V* measurements were performed inside an N_2_-filled glovebox. The curves were collected mainly with a BioLogic SP-300 potentiostat, whereas low-current regimes were measured on a Keithley 2636B source measure unit (SMU). *J*–*V* curves under simulated 1-sun illumination came from a class ABB AM1.5 G solar simulator (Oriel LCS−100, 94011 A) calibrated against an RS-ID-2 reference silicon cell (certified by Fraunhofer ISE). The solar simulator spectrum closely matched the AM1.5 G reference spectrum, and the spectral mismatch factor between the reference silicon cell and the test devices was therefore assumed to be negligible; no spectral mismatch correction was applied. Devices were masked with a 0.09 cm^2^ black aperture and scanned at 100 mV s^−1^, and no preconditioning protocol was used before measurement. For the *I*–*V* measurements shown in Extended Data Fig. 7a, obtained from the Memsol device with an integrated memristor adjacent to the solar-cell active area, the raw data were initially recorded as current–time traces and subsequently converted to *I*–*V* characteristics using the applied voltage scan rate.

### Electroluminescence measurements

Electroluminescence measurements were conducted using the BioLogic SP-300 potentiostat to apply voltage and measure current from both the device and the photodiode. A Hamamatsu S1227-1010BQ photodiode, featuring a responsivity of 0.34 A W^−1^ at 800 nm, was used to detect the emitted light. The measurement was performed inside an N_2_-filled glovebox.

### Lock-in thermography

Infrared images were acquired using a PI640i thermal camera (Optris) equipped with a 12° × 9° macro lens. The camera offers an image resolution of 640 × 480 pixels, a noise equivalent temperature difference of 0.04 K and a frame rate of 32 Hz. For the lock-in thermography images, rectangular voltage pulses with an amplitude of −0.5 V and a lock-in frequency of 1 Hz have been applied from a National Instrument source measure unit (PXIe−4138). The acquired images by the camera were demodulated according to the synchronous two-channel correlation with weighting factors that approximate a sine and a cosine function. This custom-built implemented algorithm allows us to compute the amplitude and phase of the temperature signal. For this study, we have examined the temperature amplitude image, which is proportional to the locally dissipated power that originates from the rectangular voltage pulses. For Supplementary Videos 1 and 2, the *I*–*V* measurements were performed using a Keithley 2636B SMU under the same optical setup as the camera. Measurements were conducted under ambient conditions.

### IPCE measurements

IPCE measurements were performed using a custom-built setup consisting of a halogen lamp, a monochromator and a calibrated silicon reference photodiode. The modulated light signal was detected using a lock-in amplifier synchronized to a mechanical chopper operating at a frequency of 330 Hz. The external quantum efficiency setup was calibrated with a reference silicon solar cell (RS-ID−2, FHG-ISE). The measurement was performed at ambient conditions.

### Cyclic testing

Cyclic testing was conducted using a BioLogic SP-300 potentiostat by applying different currents from −2 mA up to −40 mA, followed by monitoring the device voltage under illumination in the subsequent cycle. The measurement was performed inside an N_2_-filled glovebox. For the cyclic measurement demonstrated in Fig. 3b, each cycle comprised a 60 s dark interval under a −2 mA reverse current, followed by a 6 s illuminated open-circuit interval, while the device voltage was logged continuously. Figure 3b (top and bottom) show 50 cycles of this measurement. As before, we chose −2 mA, because this value is close to the maximum power point current of our solar cell with an area of 0.09 cm^2^. Each matching colour in the two panels represents a consecutive dark and light cycle.

### Photoluminescence and current map

Photoluminescence maps were acquired using a Horiba LabRAM HR confocal microscope and spectrometer system, using a 405 nm laser excitation at low power (about 10–20 µW) to prevent perovskite degradation during measurements. Current maps were recorded by biasing the device at 0.1 V using a Keithley 2636B SMU, controlled by a custom-developed program for real-time current acquisition.

### MPP tracking measurement of nine-cell string

A custom nine-cell holder was fabricated using a three-dimensional printer. Electrical contact to each cell was established through miniature probe pins integrated into the holder, with the probes soldered together by wiring beneath the base. For this measurement, solar cells with an enlarged single-cell active area of 0.65 cm^2^ were used, yielding a short-circuit current *I*_sc_ of approximately 16 mA. The entire setup was placed inside an N_2_-filled glovebox to prevent moisture and oxygen exposure. An array of four white LEDs positioned above the holder provided illumination and was actively cooled by a fan. The light intensity was adjusted to obtain the short-circuit current measured under the solar simulator. The measurements were taken using a Keithley 2636B Source Meter controlled by a custom Python program implementing a perturb-and-observe algorithm, with a voltage step of 0.05 V and a delay time of 0.5 s. For periodic shading experiments, a movable slider integrated into the top cover of the holder allowed selective shading and re-illumination of individual cells.

### Thermal stability measurement

Thermal stability test was carried out inside an N_2_-filled glovebox using a BioLogic SP-300 potentiostat. The Memsol and reference devices were placed on a heater maintained at 65 °C, and *I–V* characteristics were recorded periodically to monitor performance over time. For the reference cell, the bias was swept from +1.21 V to 0 V, whereas the Memsol cell sweep extended from +1.21 V to −1 V. The measurement was performed using a mask.

### Focused ion beam and scanning electron microscopy experiments

The focused ion beam (FIB) cross-section images were acquired on a TFS Helios 600i machine. To protect the sample surface from ion-beam damage during milling, a first carbon deposition was performed using the 2 kV, 0.28 nA µm^−^^2^ electron beam for 50 nm, followed by a second carbon deposition using the 30 kV, 8 pA µm^−^^2^ Ga ion beam for 500 nm. The ion-beam milling was performed using an 80 pA regular cross-section followed by a 40 pA cross-section cleaning step. The SEM imaging was performed using the TLD-SE detector in immersion mode, providing high-resolution conductivity contrast. The acquisition was performed at 2 kV, 86 pA with 0.5 nm pixel width and 5 µs dwell time. Post-processing was performed to enhance the image quality using non-local means denoising and contrast enhancement.

## Online content

Any methods, additional references, Nature Portfolio reporting summaries, source data, extended data, supplementary information, acknowledgements, peer review information; details of author contributions and competing interests; and statements of data and code availability are available at https://doi.org/10.1038/s41586-026-10275-3.

## Supplementary information

Peer Review file

Supplementary Video 1 Temperature map video of the Memsol under reverse bias.

Supplementary Video 2 Temperature map video of the reference cell under reverse bias.

## Data Availability

All data supporting the findings of this study are available within the paper, Extended Data and Supplementary Information.
