# Supplementary material for: Integrated memristor for mitigating reverse-bias in perovskite solar cells
Source: Nature. 2026 Mar 18;651(8107):933–9. doi: 10.1038/s41586-026-10275-3 (PMC13661237; doi:10.1038/s41586-026-10275-3)
Supplement: Supplementary file 1 [file 41586_2026_10275_MOESM1_ESM.pdf]

nature portfolio

Peer Review File

Integrated memristor for mitigating reverse-bias in perovskite solar cells

Corresponding Author: Professor Wolfgang Tress

Any redactions in this file are there to maintain patient confidentiality, the confidentiality of unpublished data, or to remove third-party material.

This file contains all reviewer reports in order by version, followed by all author rebuttals in order by version.

Version 0:

Reviewer comments:

Referee #1

(Remarks to the Author)
To enhance the stability of perovskite solar cells under reverse bias conditions, Mohammadi et al. introduced a novel approach called Memsol, which involves integrating a memristor into the perovskite solar cell architecture. As noted by the authors, the standard reverse-bias qualification for photovoltaic modules, outlined in IEC 61215-2:2016, includes a hot-spot endurance test under constant reverse bias to simulate partial shading and evaluate thermal robustness. I find research on the reverse-bias stability of perovskite-based solar cells particularly valuable for advancing the technology toward market readiness. However, such claims must be supported by robust evidence. Therefore, I have outlined several major questions below:
1. The authors cited the recent Nature Energy papers in the introduction but did not mention about the breakdown voltages (Vbr) of up to –15 V and –20 V in those studies. On page 1, they state: "... The inherent ionic conductivity, soft lattice structure, and susceptibility to interfacial reactions render PSCs particularly vulnerable to reverse bias stress, even at relatively low voltages ranging from -1 V to -3 V..." Given that recent works demonstrate Vbr values as high as –15 to –20 V, this statement should either be revised to reflect the current understanding or updated with specific voltage breakdown values from the cited Nature Energy examples.
2. The manuscript would benefit from a low-magnification SEM image that displays the complete Memsol structure, clearly showing both the perovskite solar cell and the integrated memristor.
3. The experimental section lacks detail regarding the deposition technique used—it is stated that PVD but please specify whether it was evaporation, sputtering, etc. What is the resolution of the shadow mask used? Furthermore, how is the active area of the device affected when converting the control solar cell to the Memsol configuration? Clarifying these points would strengthen the reproducibility and clarity of the fabrication process.
4. In Fig. S3, the authors demonstrate the reproducibility of the Memsol devices using two preparation methods for the PL177 layer, focusing on solar cell performance. However, the reproducibility of the memristor functionality itself is not addressed. The authors are encouraged to comment on the variability in memristor switching behavior across devices? If the memristor fails to operate as intended, how does this affect overall device or module performance?
5. Please include color code legends in Figure 2 to improve clarity. It would also be helpful to label the light and dark JV scans directly on the figure panels for easier interpretation.
6. A major concern is the lack of comparative data between the control (without Memsol) and Memsol devices under the same reverse bias conditions. For instance, control devices reportedly break down at around –3 to –4 V in the dark, while the Memsol device switches near –0.2 V. Given that the control device cannot endure –4 V for 1 minute, it would be useful to show how the Memsol device performs after being held at –4 V for the same duration. I can find some tests in different figures but I recommend including pre- and post-reverse bias JV curves for both control and Memsol devices to clearly illustrate their behavior. For example, Fig. 2e shows thermography of the Memsol device, but the control response is not shown. Similarly in the partial shading tests—only the Memsol device is shown, while the control's response is absent.
7. The figure 2e caption references "reverse bias" without specifying the exact applied values. Please specify the conditions for the tests.
8. It is stated in the main text that ...The IEC protocol for reverse bias is not directly applicable to the Memsol architecture, whose integrated memristor suppresses large reverse potentials by switching into a low-resistance state. For the Memsol, it is sufficient to verify that a shaded device reliably conducts the photocurrent delivered by the illuminated part of the string or

module. Upon illumination, the memristor is expected to revert autonomously to the high-resistance (off) state under the self-generated forward bias, consistent with the J-V characteristics shown in Fig. 2. Under open-circuit conditions, the Memsol voltage should therefore attain the underlying solar cell's Voc... Therefore, the partial shading experiment is critical to validate the Memsol's reliable operation. However, the current partial shading setup—only three series-connected cells—may not produce realistic reverse-bias stress. The authors are encouraged to include more devices. Even, it is important to explore perovskite Memsol mini-module testing. The reliable operation of Memsol mini-module (monolithically connected if possible- in stead of string assemble) operates under such conditions as in real life perovskite modules and comparison with reference cell would be ideal way to show the performance of Memsol device. Finally, it would be important to specify the threshold at which the Memsol structure itself fails, if any.

Referee #2

(Remarks to the Author)
This manuscript introduces a co-integrated device architecture in which a memristor is embedded alongside a perovskite solar cell to function as a bypass element under reverse-bias conditions. The concept is novel, and the experimental data convincingly demonstrate that the memristor can switch between high- and low-resistance states depending on the polarity of the applied bias. This switching behavior enables the solar cell to be protected during shading events and to recover its performance upon re-illumination. The single-cell results are clearly presented, and the fabrication process is thoroughly described.
Despite these strengths, several critical technical limitations limit the broader significance and applicability of the work.
First, the experimental validation is restricted to a single device-level measurement, with only one demonstration of a three-cell series connection. A bypass mechanism is functionally meaningful primarily in the context of large-scale photovoltaic arrays or modules, where partial shading and current mismatch are prevalent. Without validation at the array level or system-level demonstration, the proposed concept remains confined to early-stage proof-of-function.
Second, the retention behavior of the memristor is not evaluated. While switching under reverse bias and cycling stress is shown, there is no data on whether the device can maintain its resistance state under zero-bias conditions for extended durations. In practical scenarios, solar cells may remain in shaded or illuminated states for many hours, and reliable retention is crucial for long-term protection.
Third, the issue of sneak current is entirely omitted. In resistive switching arrays, unintentional current paths through neighboring devices can lead to leakage, performance degradation, or erroneous switching. This is a well-known obstacle in memristor-based systems, and without addressing it through selector layers or access transistors, the scalability and robustness of the proposed solution remain in question.
Fourth, device variability is not addressed. Resistive switching devices are known to suffer from significant cycle-to-cycle and device-to-device variation in set and reset voltages. Such variability can result in incomplete switching, false triggering, or failure to recover functionality under marginal bias conditions. Without statistical data over multiple cycles and across multiple devices, the robustness of the proposed bypass functionality remains uncertain.
Additionally, the architecture relies on lateral integration — the memristor is fabricated adjacent to the solar cell, increasing the substrate area without contributing to photocurrent. This directly reduces the power density of the device. If additional components are introduced to mitigate sneak paths, the structural complexity and area penalty would increase further. These trade-offs are not discussed, but they are essential for any realistic consideration of module-level deployment.
Finally, while the memristor is proposed as a replacement for conventional bypass diodes, the manuscript does not provide quantitative benchmarking. Key figures of merit, such as switching voltage, leakage current, thermal stability, and long-term reliability, are not compared against standard bypass elements. Without such benchmarking, it is difficult to assess whether the proposed solution offers real advantages.
In summary, this manuscript presents a creative idea with sound single-cell experiments. However, essential aspects such as array-level relevance, data retention, variability, sneak current mitigation, integration trade-offs, and benchmarking against established technologies are not sufficiently addressed. These are critical for demonstrating the viability of the approach beyond initial proof-of-concept. To establish the practical relevance of the proposed architecture, module-level demonstrations and system-level performance evaluations are essential.

Version 1:

Reviewer comments:

Referee #1

(Remarks to the Author)
The authors performed additional experiments that significantly strengthen the study. The supporting videos are beneficial and clearly demonstrate the operation of both the reference and Memsol devices under reverse bias. In its current form, the manuscript presents more reliable and comparable data for the Memsol device, and the authors now provide a detailed description of the Memsol memristor device fabrication.
One main point remains regarding the applicability of this approach to industrial processes, particularly for Method 3, which is based on lithography. It would be helpful to clarify how this method could be translated to large-scale manufacturing when we consider already existing bypass diodes. In addition, please specify which fabrication method is used for the devices discussed in the main text (if I understand correctly, it is M3). Conceptually, a discussion on this aspect will be valuable, and including a schematic illustrating how the Memsol device would be integrated or aligned within a large-area module would further improve clarity.

Additionally, I noted a few minor points:
In Figure 1F, “silver” is misspelled as “silve” and should be corrected.
Please clarify the meaning of BW and FW in Supporting Figures.
In Figure S18, the term “Mixed” likely refers to mixed SAMs, while “single” appears to refer to a single SAM; please confirm this for clarity.

Referee #2

(Remarks to the Author)
The revised submission makes an effort to respond to the earlier concerns by adding (i) a 9-cell string operated with MPPT under periodic shading, (ii) a new “limitations/scalability” section that acknowledges a reverse-current ceiling and proposes parallel memristors (including a two-memristor demonstration), (iii) additional switching statistics in the SI, and (iv) a retention measurement described in the rebuttal. These additions strengthen the laboratory-scale proof-of-concept. At the same time, they make the remaining gap to module-relevant operation more explicit, and the manuscript still does not support the breadth of its real-world practicality claims.

1. The 9-cell string/MPPT experiment remains a narrowly defined laboratory test rather than a module-representative validation. The reported conditions are highly controlled: 0.65 cm^2 cells with Isc~ 16 mA, shading of one cell roughly once per hour, 40 light–dark cycles (16 h light / 8 h dark), and MPPT paused during the 8 h dark interval. The string operates around ~12 mA and ~8–9 V. The Methods/SI further indicate nitrogen glovebox testing with LED illumination and fan cooling. This is a useful engineering sanity check, but it does not address the regimes that will determine whether a bypass strategy is viable in practice (higher operating currents, encapsulation constraints, temperature swings, and realistic, sharply varying shading dynamics).

2. The revision now openly states a hard current-handling constraint that appears incompatible with realistic PV operation unless a large parallel network is used, and the manuscript does not convincingly validate that scaling route. The new text argues that (Voc) limits the reset voltage and therefore the allowable reverse current, which “should remain below –40 mA.” It also acknowledges that earlier demonstrations were effectively limited to ~2 mA by the small cell area, while practical cells are “100s of cm^2.” Under those stated conditions, deploying many memristive elements in parallel (and at the stated density of ~1–2 per cm^2) is not a minor implementation detail; it is central to feasibility. The manuscript does not provide evidence that a large population of parallel elements will switch deterministically and share current in a stable, controlled way over time and cycling.

3. Even the simplest parallel demonstration shows non-simultaneous behavior, which challenges the premise that parallel memristors “always switch together” without independent addressing. In the revised text, the delayed turn-on of the second device is described as “slightly delayed,” while the rebuttal frames simultaneous switching as intended and required for parallelization. What is missing—yet essential for scalability—is quantitative reporting: how large the delays are, how broadly they vary device-to-device, how they depend on cycling/history, and whether transient current hogging occurs during those delays. In large parallel networks, even a small fraction of late/early switchers can dominate instantaneous stress and reliability, so this behavior needs to be characterized rather than described qualitatively.

4. The revision reports non-ideal state behavior that would reduce delivered voltage/power, but it is not quantified in a way that allows readers to judge the impact. The manuscript states that in illumination cycles 10, 12, and 22, the memristor initially switched off but subsequently turned on again, causing a temporary reduction in (Voc) before switching off again. The caption also notes cases where (Voc) “has not been reached.” These are not cosmetic waveform features; they imply that the bypass element can enter (and re-enter) states that reduce string output. The manuscript should summarize how often these events occur across the dataset, typical recovery times, and the resulting energy-yield penalty (even a simple Wh estimate over the test sequence would be informative).

5. The rebuttal’s position on retention and bias conditions is not clearly reconciled with the revised operating protocol, and the resolution is not fully anchored in the archival manuscript/SI. The rebuttal argues retention is “not a required property” because the device is continuously biased, and that true zero-bias intervals occur only when the array is idle. However, the revised MPPT protocol explicitly includes an 8-hour dark interval where MPPT tracking is paused. The manuscript does not state what electrical boundary condition the MemSol actually sees during this paused period (true zero-bias, floating, held bias, or something else). Because the “retention not required” argument depends on continuous bias, the paper should clearly state the actual bias condition during the dark/paused interval, so the operational rationale is internally consistent. Separately, if a retention test (~380 h under periodic reads) is being offered as evidence, it should be straightforward to locate in the SI as part of the archival record (clearly labeled and easy to find), rather than being primarily described in rebuttal text.

6. The bypass-diode benchmarking is performed in the low-current regime, where the authors themselves expect the memristor to appear favorable, but an equivalent comparison near the stated higher-current limit is not shown. The manuscript compares against a 1N5819 and reports ~0.4 V loss for the diode versus <0.1 V for the MemSol, with an illustrative power-loss comparison. Yet the rebuttal also states that the memristor’s on-voltage is lower than diodes, mainly below ~10 mA, while around ~40 mA, its on-voltage becomes comparable, one of the motivations for parallelization. Given that the string test runs at ~12 mA, the revision still does not demonstrate comparable (or superior) dissipation near the authors’ own current ceiling, nor does it show that a parallel-memristor configuration delivers the claimed advantage at those currents.

7. The rebuttal asserts applicability to an “arbitrary number” of series-connected cells, but the revised manuscript introduces explicit current constraints and a parallelization requirement that are not reconciled with that statement. A module-level argument would need to map realistic reverse-current distributions under partial shading onto the −40 mA constraint, then show how the required parallel network can be implemented without introducing the non-simultaneity/current-sharing issues that are already visible even with two devices.

8. Independent of the technical details, the overall scope remains misaligned with what this journal typically expects when broad claims are made about module-level practicality. What is demonstrated is a controlled, low-current, small-area proof-of-concept. The path to practical relevance relies on large parallel networks per cell, yet the key behaviors that would make such networks viable—deterministic synchronized switching and robust current sharing—are not established even in the simplest parallel case. In its current form, the work appears better suited to a more specialized venue focused on early-stage device concepts or memristor physics.

Overall, the revision shows that the concept can function in a controlled laboratory string test. However, the newly added “limitations/scalability” discussion and the observed non-ideal switching behaviors reinforce that practical deployment would require large parallel networks, and that synchronized switching/current sharing—central to that approach—is not demonstrated. The mismatch between the demonstrated operating regime and what real PV modules demand remains unresolved in the revised manuscript.

Open Access This Peer Review File is licensed under a Creative Commons Attribution 4.0 International License, which permits use, sharing, adaptation, distribution and reproduction in any medium or format, as long as you give appropriate credit to the original author(s) and the source, provide a link to the Creative Commons license, and indicate if changes were made.
In cases where reviewers are anonymous, credit should be given to 'Anonymous Referee' and the source.
The images or other third party material in this Peer Review File are included in the article’s Creative Commons license, unless indicated otherwise in a credit line to the material. If material is not included in the article’s Creative Commons license and your intended use is not permitted by statutory regulation or exceeds the permitted use, you will need to obtain permission directly from the copyright holder.
To view a copy of this license, visit https://creativecommons.org/licenses/by/4.0/

Dear editor,

We sincerely thank the reviewers for their thorough evaluation and constructive feedback, which have greatly helped us improve the quality and clarity of the manuscript. In response to the reviewers' comments, we have made several substantial revisions and additions, summarized below:

1. Integration of the Memristor into the active area:

   Whereas in the original version the memristor has been placed adjacent to the photovoltaically active area, we have integrated the memristor directly within the active area of the solar cell. All measurements (except Fig. 2E) have been re-performed using this new fully-integrated device architecture.

2. Cross-sectional SEM Image:

   A new low-magnification cross-sectional SEM image illustrating the complete Memsol structure, including both the perovskite solar cell and the integrated memristor, has been added to the Supplementary Information to better visualize the device configuration.

3. Photocurrent–PL Mapping:

   We have included simultaneously measured photocurrent and photoluminescence (PL) maps to examine the influence of memristor integration within the solar cell active area. These results confirm that the memristor footprint (<1 % of the active area) is negligible on photocurrent and performance.

4. Statistical Analysis of Switching Behavior and Reliability:

   New statistical datasets have been incorporated to demonstrate the reproducibility and reliability of the Memsol switching behavior under various measurement conditions and current stress levels, also discussing the limitations and providing the solution of multiple memristor integration, important for upscaling.

5. Extended MPP Tracking under Partial Shading:

   To approximate real-world operating conditions, we performed maximum power point (MPP) tracking measurements on a string of nine series-connected cells (each with an active area of 0.65 cm^2) under continuous illumination, with one cell periodically shaded to simulate dynamic partial-shading events. This approach effectively replicates the electrical behavior of a small-scale module. The results, presented in Fig. 6, confirm that the Memsol maintains stable performance and effectively suppresses reverse bias during extended shading cycles.

Referee #1 (Remarks to the Author):

To enhance the stability of perovskite solar cells under reverse bias conditions, Mohammadi et al. introduced a novel approach called Memsol, which involves integrating a memristor into the perovskite solar cell architecture. As noted by the authors, the standard reverse-bias qualification for photovoltaic modules, outlined in IEC 61215-2:2016, includes a hot-spot endurance test under constant reverse bias to simulate partial shading and evaluate thermal robustness. I find research on the reverse-bias stability of perovskite-based solar cells particularly valuable for advancing the technology toward market readiness. However, such claims must be supported by robust evidence. Therefore, I have outlined several major questions below:

We are glad to read that the reviewer regards our efforts on solving the reverse-bias stability challenge as valuable and have addressed all their comments as described point-by-point in the following.

1. The authors cited the recent Nature Energy papers in the introduction but did not mention about the breakdown voltages (Vbr) of up to –15 V and –20 V in those studies. On page 1, they state: "… The inherent ionic conductivity, soft lattice structure, and susceptibility to interfacial reactions render PSCs particularly vulnerable to reverse bias stress, even at relatively low voltages ranging from -1 V to -3 V…" Given that recent works demonstrate Vbr values as high as –15 to –20 V, this statement should either be revised to reflect the current understanding or updated with specific voltage breakdown values from the cited Nature Energy examples.

The statement in the Introduction has been revised to reflect the broader range of reported breakdown voltages in recent literature, including the Nature Energy examples already cited. The revised text now acknowledges that while perovskite solar cells can exhibit breakdown at relatively low voltages (–1 V to –3 V) depending on interface and material quality, several recent studies have reported much higher breakdown thresholds, up to –15 V to –20 V, under specific device architectures and testing conditions.

2. The manuscript would benefit from a low-magnification SEM image that displays the complete Memsol structure, clearly showing both the perovskite solar cell and the integrated memristor.

A low-magnification cross-sectional SEM image illustrating the complete Memsol structure, including both the perovskite solar cell and the integrated memristor, has now been added to the Supplementary Information (Fig. S3). The higher-magnification images presented in Fig. 1C and 1F are cropped from this newly included overview image.

3. The experimental section lacks detail regarding the deposition technique used—it is

stated that PVD but please specify whether it was evaporation, sputtering, etc. What is the resolution of the shadow mask used? Furthermore, how is the active area of the device affected when converting the control solar cell to the Memsol configuration? Clarifying these points would strengthen the reproducibility and clarity of the fabrication process.

The Experimental Section has been revised accordingly. Specifically, the PL177 layer was deposited by physical vapor deposition (PVD) using thermal evaporation, and the shadow mask resolution employed for evaporation was 50 μm.

To demonstrate that the integration of the memristor hardly reduces the effective photovoltaic area, we have added simultaneously measured photocurrent and photoluminescence (PL) maps to the revised manuscript (Fig. 2F and 2G). These maps confirm that the footprint of the integrated memristor occupies less than 1 % of the solar cell's active area, thereby having a negligible impact on the measured current densities and overall device performance. In fact, we did not observe any noticeable change in the measured photocurrent.

4. In Fig. S3, the authors demonstrate the reproducibility of the Memsol devices using two preparation methods for the PL177 layer, focusing on solar cell performance. However, the reproducibility of the memristor functionality itself is not addressed. The authors are encouraged to comment on the variability in memristor switching behavior across devices? If the memristor fails to operate as intended, how does this affect overall device or module performance?

To address this, we have now included statistical data from ten independent Memsol devices (see Fig. 3D) showing the distribution of switching voltage and open-circuit voltage (V_{oc}) over 400 consecutive dark–light cycles. In addition, we provide a second dataset (Fig. 4B) comparing the switching voltage and V_{oc} of Memsol devices under varying and higher reverse current limits than –2 mA, thereby illustrating the robustness of the switching behavior across different stress conditions.

In terms of failure modes, the Memsol's turn-on process is highly reliable, as any sufficiently high negative bias beyond the threshold inevitably activates the memristor. The turn-off process, governed by the self-generated forward bias under illumination, may occasionally exhibit delayed response. As demonstrated in Fig. 3B, 3F, and 4B, prolonged illumination consistently restores the high-resistance (off) state. In rare cases where the memristor remains latched in the low-resistance state for longer time, it effectively forms a temporary shunt-thus still protecting the solar cell from reverse-bias damage, although no output power is then delivered from that particular cell.

5. Please include color code legends in Figure 2 to improve clarity. It would also be helpful to label the light and dark JV scans directly on the figure panels for easier interpretation.

We have adjusted the figure as suggested.

6. A major concern is the lack of comparative data between the control (without Memsol) and Memsol devices under the same reverse bias conditions. For instance, control devices reportedly break down at around –3 to –4 V in the dark, while the Memsol device switches near –0.2 V. Given that the control device cannot endure –4 V for 1 minute, it would be useful to show how the Memsol device performs after being held at –4 V for the same duration. I can find some tests in different figures but I recommend including pre- and post-reverse bias JV curves for both control and Memsol devices to clearly illustrate their behavior. For example, Fig. 2e shows thermography of the Memsol device, but the control response is not shown. Similarly in the partial shading tests—only the Memsol device is shown, while the control's response is absent.

The reason for the lack of comparative data is that the switching of the Memsol to a low-resistance state would never allow for a voltage of e.g. -4 V under realistic operation, since the current provided by the series-connected solar cells would not be sufficient (given that sufficient memristive elements are integrated in parallel for a given solar cell area, see below). Nevertheless, we performed the same measurement for Memsol and Reference, to -4V, considering that the current cannot exceed the photocurrent of our solar cells, which we set as a compliance current. We have added IV curves as Fig. 3A and see that the Memsol reaches the compliance current at low reverse voltage. Thus, any number of solar cells connected in series would not be able to harm the Memsol, whose protective functionality relies on the switching behavior, which effectively prevents the occurrence of large reverse voltages that would otherwise degrade the solar cell. We have also added the IV curves under light before and after this measurement (Fig. S17), allowing for a direct comparison of Memsol and reference.

Therefore, in the Memsol configuration, the relevant stress parameter is the reverse current, not the voltage, since the integrated memristor limits the reverse potential by switching into a low-resistance state that safely conducts the photocurrent. Thus, we added measurements regarding the maximum current that can be tolerated by the Memsol, which would define how many memristive elements are needed per photoactive area. Fig. 4A demonstrates that the memristor can sustain reverse currents up to 40 mA while the set voltage remains below approximately –1 V.

We have included a thermography map of the active-area integrated memristor in the main manuscript (Fig. 2D) and moved the one of the memristor adjacent-to-active area to the SI. We have also added a thermography map of the reference in the SI (Fig. S13).

We have also performed the partial shading test for the reference and for a configuration with external bypass diodes and added the discussion to the respective parts of the manuscript (see point no. 8).

We have also added Fig. 3A, and Fig. S17 which presents the pre- and post-stress J–V characteristics for both the reference and Memsol devices measured under identical conditions.

7. The figure 2e caption references "reverse bias" without specifying the exact applied values. Please specify the conditions for the tests.

The figure has been updated.

8. It is stated in the main text that ...The IEC protocol for reverse bias is not directly applicable to the Memsol architecture, whose integrated memristor suppresses large reverse potentials by switching into a low-resistance state. For the Memsol, it is sufficient to verify that a shaded device reliably conducts the photocurrent delivered by the illuminated part of the string or module. Upon illumination, the memristor is expected to revert autonomously to the high-resistance (off) state under the self-generated forward bias, consistent with the J-V characteristics shown in Fig. 2. Under open-circuit conditions, the Memsol voltage should therefore attain the underlying solar cell's Voc... Therefore, the partial shading experiment is critical to validate the Memsol's reliable operation. However, the current partial shading setup—only three series-connected cells—may not produce realistic reverse-bias stress. The authors are encouraged to include more devices. Even, it is important to explore perovskite Memsol mini-module testing. The reliable operation of Memsol mini-module (monolithically connected if possible- in stead of string assemble) operates under such conditions as in real life perovskite modules and comparison with reference cell would be ideal way to show the performance of Memsol device. Finally, it would be important to specify the threshold at which the Memsol structure itself fails, if any.

We understand the reviewer's suggestion regarding extended partial-shading and mini-module testing. To better approximate realistic operating conditions, we constructed a string mini-module consisting of nine series-connected cells with an enlarged active area of 0.65 cm^2 per cell, effectively emulating the electrical behavior of a small-scale module.

We have now included new maximum power point (MPP) tracking measurements performed on this nine-cell string under continuous illumination, during which one cell was periodically shaded to simulate dynamic partial-shading conditions. These measurements were designed to assess the long-term stability and self-recovery behavior of the Memsol during continuous operation. The results, now presented in Fig. 6, demonstrate that the Memsol maintains stable performance and effectively suppresses reverse bias during shading events, in exactly the same manner as if 3 (previous data) devices have been connected in series. Given this indifferent behavior and its working principle, it will also work for an arbitrary number of serially connected solar cells.

For a comprehensive comparison, the same experiment was repeated with (i) a reference string containing a standard perovskite cell in place of the Memsol and (ii) a reference string with three cells protected by a conventional bypass diode. This enables direct evaluation of the Memsol's reliability and protective function relative to established bypass strategies.

Referee #2 (Remarks to the Author):

This manuscript introduces a co-integrated device architecture in which a memristor is embedded alongside a perovskite solar cell to function as a bypass element under reverse-bias conditions. The concept is novel, and the experimental data convincingly demonstrate that the memristor can switch between high- and low-resistance states depending on the polarity of the applied bias. This switching behavior enables the solar cell to be protected during shading events and to recover its performance upon re-illumination. The single-cell results are clearly presented, and the fabrication process is thoroughly described. Despite these strengths, several critical technical limitations limit the broader significance and applicability of the work.

We also thank reviewer 2 for pointing out the strengths and have been working to address the limitations in the revised version.

First, the experimental validation is restricted to a single device-level measurement, with only one demonstration of a three-cell series connection. A bypass mechanism is functionally meaningful primarily in the context of large-scale photovoltaic arrays or modules, where partial shading and current mismatch are prevalent. Without validation at the array level or system-level demonstration, the proposed concept remains confined to early-stage proof-of-function.

We fully understand that a three-cells demonstration is not regarded sufficient. As already discussed in response to reviewer 1 point 8, we have extended the number of cells and their area. Based on these results and the working mechanism of the Memsol, we can safely say that it will also work in larger assemblies.

Second, the retention behavior of the memristor is not evaluated. While switching under reverse bias and cycling stress is shown, there is no data on whether the device can maintain its resistance state under zero-bias conditions for extended durations. In practical scenarios, solar cells may remain in shaded or illuminated states for many hours, and reliable retention is crucial for long-term protection.

In practical operation, the Memsol is continuously biased - either by a reverse voltage and current under dark (if it is shaded and the other cells in series are under illumination) or

by the photovoltaically self-generated forward bias under illumination. Thus, retention is not a required property. When zero-bias conditions are reached, the whole solar-cell array would be idle and the state of the memristor would not matter.

Nevertheless, to address the reviewer's concern, we have conducted an additional retention test in which the Memsol was first switched to the ON state by applying reverse bias in dark and subsequently monitored under a small negative read voltage to evaluate the duration for which it can preserve its low-resistance state. The results confirm that the Memsol remains in the ON state for approximately 380 hours before reverting to the OFF state.

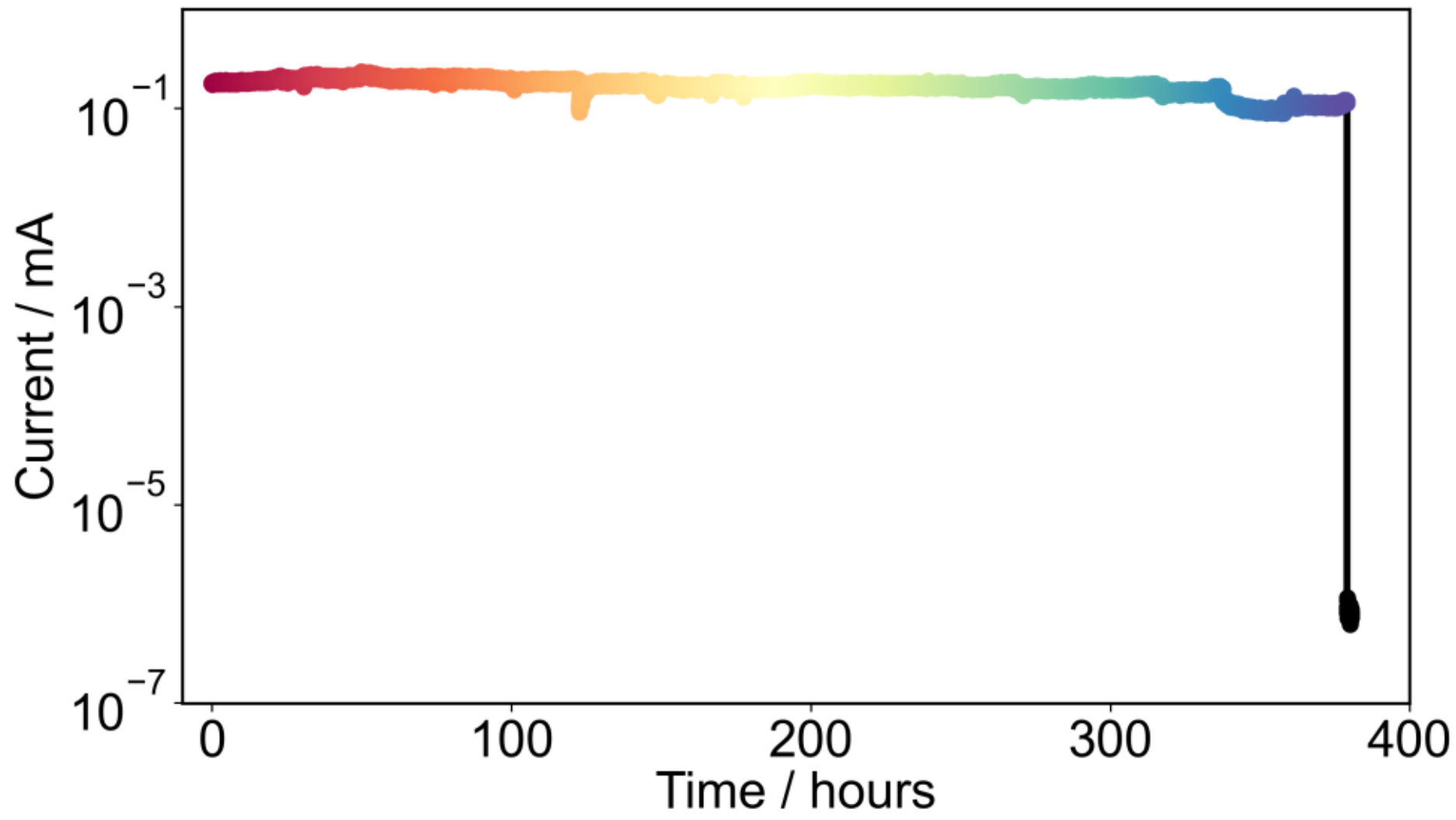

Figure 1. Retention behavior of the Memsol device measured under a constant read bias of –0.05 V DC read pulse (0.1 s duration) applied every 10 s. The current remains stable in the ON state for approximately 380 hours before reverting to the OFF state, confirming long-term retention stability under low-bias conditions.

Third, the issue of sneak current is entirely omitted. In resistive switching arrays, unintentional current paths through neighboring devices can lead to leakage, performance degradation, or erroneous switching. This is a well-known obstacle in memristor-based systems, and without addressing it through selector layers or access transistors, the scalability and robustness of the proposed solution remain in question.

Sneak currents are not relevant in the present Memsol configuration, as our architecture incorporates only one memristor per solar cell rather than a crossbar array in which individual memristors must be selectively addressed. The next memristor would be in the adjacent separated and just serially connected solar cell, such that leakage currents cannot form. In case of larger photoactive areas, several memristors are needed for one solar cell (as we show in the revised version). These memristors are connected in parallel and are designed and required to always switch simultaneously, being either ON, when the solar cell is shaded, or OFF, when it is under light. Selector layers are not needed either,

since the resistance of the solar cell in parallel to the memristor automatically takes this role, Consequently, no independent addressing of the integrated memristors is required.

Fourth, device variability is not addressed. Resistive switching devices are known to suffer from significant cycle-to-cycle and device-to-device variation in set and reset voltages. Such variability can result in incomplete switching, false triggering, or failure to recover functionality under marginal bias conditions. Without statistical data over multiple cycles and across multiple devices, the robustness of the proposed bypass functionality remains uncertain.

We have now included statistical data collected from ten independent Memsol devices (see Fig. 3D) and over 400 dark light switching cycles (see Fig. 4B). These results demonstrate consistent switching voltages, confirming the reproducibility and robustness of the memristive bypass functionality across multiple devices and operating cycles.

Additionally, the architecture relies on lateral integration — the memristor is fabricated adjacent to the solar cell, increasing the substrate area without contributing to photocurrent. This directly reduces the power density of the device. If additional components are introduced to mitigate sneak paths, the structural complexity and area penalty would increase further. These trade-offs are not discussed, but they are essential for any realistic consideration of module-level deployment.

We fully agree with the reviewer that the issue of area penalty needs to be addressed. One motivation for having the memristor adjacent to the solar cell could be that it could use part of the photovoltaically "dead" area in a monolithic module. In the revised manuscript, we have the memristor successfully and fully integrated within the solar cell's active area using a lithography-based process, rather than being placed laterally adjacent to it (see new thermography map in Fig. 2D). This allows us to directly compare and quantify how much photoactive area is lost. From the current-voltage measurements of the devices we were not able to see a clearly measurable decrease in current. We then mapped the photocurrent and quantified the area that does not contribute to the photocurrent (see Fig. 3F and 3G), which is already less than 1 % of the active area of our small test cells and can be reduced by a further factor of 5 to 10, before a second memristor has to be added (see Fig. 4). This means that its effect on the effective power density of the device is negligible.

Finally, while the memristor is proposed as a replacement for conventional bypass diodes, the manuscript does not provide quantitative benchmarking. Key figures of merit, such as switching voltage, leakage current, thermal stability, and long-term reliability, are not

compared against standard bypass elements. Without such benchmarking, it is difficult to assess whether the proposed solution offers real advantages.

We have now included new maximum power point (MPP) tracking measurements performed on this nine-cell string under continuous illumination, during which one cell was periodically shaded to simulate dynamic partial-shading conditions (Fig.6)

For a comprehensive comparison, the same experiment was repeated with (i) a reference string containing a standard perovskite cell in place of the Memsol and (ii) a reference string with three cells protected by a conventional bypass diode. This enables direct evaluation of the Memsol's reliability and protective function relative to established bypass strategies.

The memristor exhibits a significantly lower turn-on voltage for currents below 10 mA, while at higher currents (around 40 mA) it achieves an on-voltage comparable to that of standard bypass diodes. Importantly, the leakage current of a single memristor element is approximately three orders of magnitude lower than that of a commercial bypass diode, and the total current-handling capability can be adjusted by connecting several memristors in parallel, if required.

In terms of thermal stability, the measurements presented in Fig. 5 indicate that the integration of the memristor within the active region does not limit the overall stability of the solar cell. The Memsol device exhibits the same degradation rate as the reference cell at 65 °C, confirming that the memristor itself remains thermally stable under operating conditions. Therefore, the thermal stability of the Memsol is governed by the solar cell component rather than by the memristor.

As discussed in the revised text, the main advantage of the integrated memristor lies in its ability to reduce both cost and power loss. In conventional module architectures, a single bypass diode typically protects multiple cells, meaning that shading of even one cell can lead to power loss from the entire string. In contrast, each Memsol cell incorporates its own memristive bypass element, ensuring that only the shaded cell is bypassed, while the remaining cells continue to operate at their maximum power point. This cell-level protection not only improves energy yield under partial shading but also reduces system cost by eliminating the need for external bypass diodes and additional wiring. The table below summarizes the key parameters characterizing the bypass functionality of the conventional bypass diode and the integrated Memsol.

Table1. A comparison of the key parameters defining the bypassing functionality of the conventional bypass diode and the integrated memristor.

Parameter	Bypass diode	Memsol
Off-state resistivity	Typically, in the range of 10^{6}–$10^{7}\,\Omega$ (limited by diode reverse leakage)	Typically, exceeding $10^{9}\,\Omega$, minimizing leakage current and reverse-bias losses
On-state resistivity	0.05 ohm -2 ohm	Few ohms (sufficiently low for mA–level currents)
Switching threshold	~0.6 – 0.7 V (intrinsic to the Si junction)	~0.035 – 0.05 V (field-induced resistive switching), minimizing power losses
Switching Speed	≈ 30 µs (junction-limited)	< 50 ns
Number of cells protected by each protective element	Typically one diode per 18–24 cells in commercial modules to minimize cost and wiring complexity.	One or multiple memristors integrated in each cell (fully distributed protection).
Power loss determined by the number of cells per protective element When a single cell within a protected string becomes shaded [1].	Power from the entire protected string is lost, in addition to the power dissipated in the bypass diode.	Only the shaded cell is isolated; all other cells in the string remain active, minimizing power loss.

[1] For a 20-cell series string at V_{mpp}=1 V and I_{mpp}=20 mA, a conventional bypass diode with a 0.7 V turn-on dissipates 14 mW when one cell is shaded, and the entire string's 0.4 W output is lost (total loss ≈ 0.414 W), whereas for a 20-cell string of Memsol devices with a 0.3 V clamp the Memsol itself dissipates only 7 mW and only the shaded cell's 0.02 W is lost (total loss ≈ 0.027 W).

In summary, this manuscript presents a creative idea with sound single-cell experiments. However, essential aspects such as array-level relevance, data retention, variability, sneak current mitigation, integration trade-offs, and benchmarking against established technologies are not sufficiently addressed. These are critical for demonstrating the viability of the approach beyond initial proof-of-concept. To establish the practical relevance of the proposed architecture, module-level demonstrations and system-level performance evaluations are essential.

We believe that we made a further step towards a more practical proof of concept and hope that the reviewer and editor agree that we have reached the level required for publication.
